# Fundamental Scaling Laws in Nanophotonics

**DOI:** 10.1038/srep37419

**Published:** 2016-11-21

**Authors:** Ke Liu, Shuai Sun, Arka Majumdar, Volker J. Sorger

**Affiliations:** 1 Department of Electrical and Computer Engineering, The George Washington University, Washington, D.C. 20052, USA; 2 The Key Laboratory of Optoelectronics Technology, Ministry of Education, Faculty of Information Technology, Beijing University of Technology, Beijing 100124, P.R. China; 3 Department of Electrical Engineering, University of Washington, Seattle, WA 98195, USA; 4 Physics Department, University of Washington, Seattle, WA 98195, USA

## Abstract

The success of information technology has clearly demonstrated that miniaturization often leads to unprecedented performance, and unanticipated applications. This hypothesis of “smaller-is-better” has motivated optical engineers to build various nanophotonic devices, although an understanding leading to fundamental scaling behavior for this new class of devices is missing. Here we analyze scaling laws for optoelectronic devices operating at micro and nanometer length-scale. We show that optoelectronic device performance scales non-monotonically with device length due to the various device tradeoffs, and analyze how both optical and electrical constrains influence device power consumption and operating speed. Specifically, we investigate the direct influence of scaling on the performance of four classes of photonic devices, namely laser sources, electro-optic modulators, photodetectors, and all-optical switches based on three types of optical resonators; microring, Fabry-Perot cavity, and plasmonic metal nanoparticle. Results show that while microrings and Fabry-Perot cavities can outperform plasmonic cavities at larger length-scales, they stop working when the device length drops below 100 nanometers, due to insufficient functionality such as feedback (laser), index-modulation (modulator), absorption (detector) or field density (optical switch). Our results provide a detailed understanding of the limits of nanophotonics, towards establishing an opto-electronics roadmap, akin to the International Technology Roadmap for Semiconductors.

Electronic device scaling has been extensively investigated with respect to geometry, power consumption, and switching speeds^1^. While scaling allows transistors with increased drive current and switching speed, it also enables denser integration resulting in versatile chip functionality and monetary incentives. Upon aggressive scaling, fundamental challenges however are found with respect to gate leakage, drain induced barrier lowering for transistors, and parasitic capacitances at the circuit level, leading to the demise of “Moore’s law”^2^. The latter opens opportunities for optics since optical ‘wires’, i.e., waveguides, are not limited by electrical capacitance^3^,^4^. In addition, the parallelism offered by the bosonic nature of photons such as explored in wavelength-division-multiplexing, has made opto-electronics attractive for communication and interconnect applications^5^. While the fundamental prospects for photonics are well-known compared to electronic devices, the diffraction limit of light poses a serious constrain for photonics to be footprint-density competitive^6^. In fact, this limitation demands a far superior performance of optical devices compared to its electronic counter-parts. For example, the typical size of electronic devices is about 20 nm, and photonic diffraction limit is λ/2*n* ~ 200 nm, where λ is the telecommunication optical wavelength, and *n* is the refractive index of the waveguide (e.g., Silicon, *n* = 3.5). Hence, clearly the optical devices need to perform at least two orders of magnitude higher compared to electronics. To faithfully reflect this trade-off between areal footprint and performance, we introduce a new Figure of Merit, FOM = [Speed/(Energy/bit × Footprint)] to assess the quality of an optoelectronic link performance. Thus, a larger FOM can be achieved by either increasing the operations per second, lowering the energy, or by reducing the area footprint. The device insertion loss is defined for the link-level, where the particular device-to-waveguide connection becomes important. In the link analysis below, the laser power is calculated based on the minimum current required by the next stage the link is connected to, while compensating all the insertion losses from both devices and waveguide sections. Nominal insertion loss values from the literature are used here, since optimizing the insertion loss is an engineering task being sensitive to material and process variations.

With technology options such as plasmonics^7^,^8^,^9^, nanoscale dielectric resonators^10^,^11^ and slot-waveguides^12^, opportunities exist to surpass the diffraction limit of light by engineering the effective refractive index. However, decreasing the optical mode volume, *V*_*m*_, introduces adverse effects, for example, bending losses, and ohmic losses for polaritonic modes. It is therefore not straightforward to predict optoelectronic performance for device scaling into the nanoscale^13^,^14^, and a rigorous analysis of fundamental scaling laws for nanophotonics as a function of critical device length is warranted. Here, we investigate the performances of four actively-controlled (electrically or optically) devices with respect to their scaling behavior: a light source, an electrical-to-optical (EO) data encoder typically in form of an electro-optic modulator, and a photodetector for the inverse OE conversion, along with a fourth device, which is a purely optical switch for all-optical information processing (Fig. 1a). We investigate their performance in terms of speed, energy and the newly introduced FOM.

In our analysis, we assume that underlying these devices are three types of optical cavities, (a) a traveling-wave ring resonator (RR)^15^, (b) a metal-mirror based Fabry-Perot (FP) cavity^16^, and (c) a plasmonic metal nano-particle (MNP)^17^ (Fig. 1b), that enhance the fundamentally weak interaction between light and matter via the ratio of *Q/V*_*m*_, where *Q* is the cavity quality factor, *V*_*m*_ is the effective volume of electromagnetic energy of a resonant mode. An interesting, although expected result is, that all cavity types do not perform equally well for vanishing critical dimension due to their respective non-monotonic Purcell factor.

For our scaling law analysis we define the critical length for the three underlying cavities as the radius for the RR and the MNP, and the physical distance between two mirrors for the FP. We derive analytical expressions for both the cavity quality factor *Q* and the optical mode volume *V*_*m*_ for the RR and FP cavities and estimate the Purcell factor (Fig. 2), defined as^18^ 
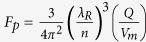
, where *λ*_*R*_ is the resonant wavelength of the cavity, and *n* is the cavity material refractive index. The discontinuity of the displacement current across the metal particle in the plasmonic cavity requires a different approach; we find the ratio of the effective density of the surface plasmon modes, *ρ*_*SP*_, relative to that of the radiation continuum, *ρ*_*rad*_, which directly gives *F*_*p*_^17^. The mode volume *V*_*m*_ is obtained by a permittivity-modified geometric volume^19^, from which we can estimate *Q*. An abbreviated equation set for the Purcell factor of the three cavities is summarized in the methods section below, whereas a detailed device performance analysis along with a comprehensive set of details of this manuscript is reported in the supplementary online material (SOM)^20^.

Regarding cavity performance, *Q* as a function of the critical length is a key metric since it relates the ability to spectrally store optical energy relative to its loss (Fig. 2a). For the RR, at larger length, *Q* is almost independent of length, since the increased propagation loss with the circumference of RR is cancelled by the increased round-trip time. However, at small radius (<10 μm), bending losses overcome the propagation losses resulting in a sharp drop in *Q*. For the FP and MNP, *Q* decreases with scaling, mainly due to an increased overlap of the electromagnetic field with the lossy metal. While the optical mode volume *V*_*m*_ of the RR can fundamentally scale with *V*_*m*_ ~ *r*^*α*^, where *α* = 1, 2, or 3 depending on how many dimensions are being scaled, fabrication and integration constraints render quadratic and cubic scaling impractical due to mode matching incompatibility issues such as multi vs. single mode ring-to-bus coupling. We therefore choose a lowest-volume approach by selecting the single-mode condition for the ring waveguide cross-section dimension and scale only the ring radius for the RR, whereas the MNP scales cubically, *V*_*m*_ ~ *a*^3^ (Fig. 2b). For the FP, the mode-volume has a non-monotonic variation with the critical length; upon scaling and approaching the diffraction limit (~200 nm), *V*_*m*_ decreases. However, with further scaling, *V*_*m*_ increases as its mode character changes; the propagation direction of the confined mode changes from longitudinal to transverse due to the formation of a metal-insulator-metal (MIM) mode where the cavity reflection is not governed by the metallic mirrors any more, but by the localized modal MIM distribution^20^,^21^.

Based on the above analysis, we investigate the scaling behavior of the Purcell factor for each cavity (Fig. 2c). For all the three cavities, we observe a non-monotonic dependence of *F*_*p*_ with scaling: for longer lengths an increased *V*_*m*_ reduces the Purcell factor, whereas at short lengths the reduction in *Q* is detrimental to *F*_*p*_. However the optimal cavity length and the maximum Purcell factor for each cavity are vastly different; the RR can provide the highest Purcell factor among all the cavities (>10^3^) provided *Q* exceeds 10^5^ and real estate of ten’s of microns for the radius are available. On the other hand, a maximum *F*_*p*_ of 10^2^ at lengths of just 10 nm is achievable in MNP as *Q* reduces slower than *V*_*m*_ does. The FP can provide a moderate *F*_*p*_ ~ 50, for a moderate length of ~10 *μm*. We also see that each cavity geometry is limited to a minimum possible length; with the assumption of the RR-forming waveguide being diffraction limited, a geometrical limit of the RR radius is reached at a radius equal to half of the diffraction limit, where the ring becomes a disc. While the minimal length of the FP can approach zero, the practical limit is near the diffraction limit since the cavity changes its mode from diffraction limited to a metal-insulator-metal gap mode, as described earlier. While the MNP has no cut-off length, the mode becomes unfeasible at atomic scales where quantum effects become dominant^22^. Hence lengths of a few nanometers are the minimum considered here^17^. In the remainder of the paper, we apply these cavity-based scaling laws to four opto-electronic devices, namely, lasers, modulators, photodetectors, and all-optical switches, and extract key information to provide both device and interconnect link FOMs.

## Laser

**Figure 2:**
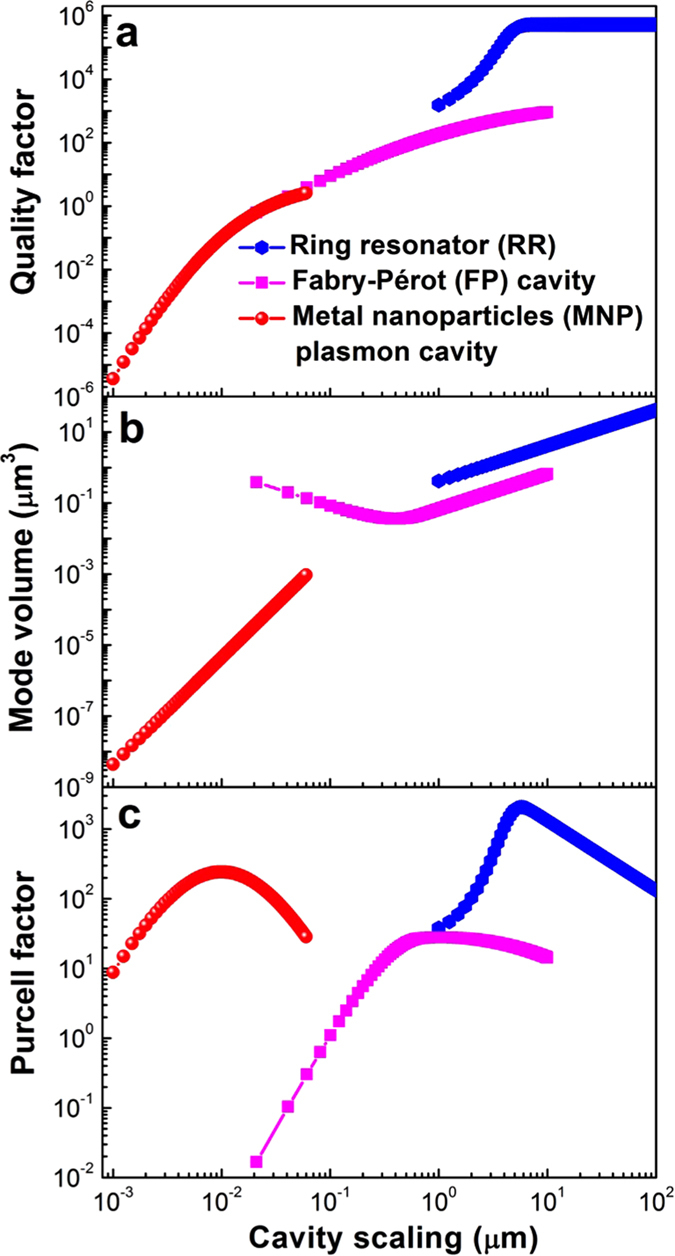
Cavity performance as a function of scaling. Scaling of (**a**) quality factor *Q*, (**b**) mode volume, *V*_*m*_ and (**c**) Purcell factor, *F*_*p*_, for all three cavity configurations. While the general trend shows a reduced *Q* upon scaling, significant differences between the three cavity types exist. Nominal parameters in this study are as follows; the propagation loss of a diffraction limited beam, *α*_*p*_ = 1.0 dB/cm such as used in the RR; Silver metal mirrors, 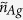 = 0.41 + 10.05i, the dielectric refractive index is taken to be 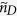 = 3.0 − i0.001, the Silver conductivity *σ*_*Ag*_ = 6.3 × 10^7^ mho/m, the dipole distance from the MNP to be *d* = 10 nm, and the damping rate for the MNP to be *γ*_*d*_ = 2.0 × 10^15^ rad/s. Clear maxima upon scaling are observed for all three cavities.

**Figure 3:**
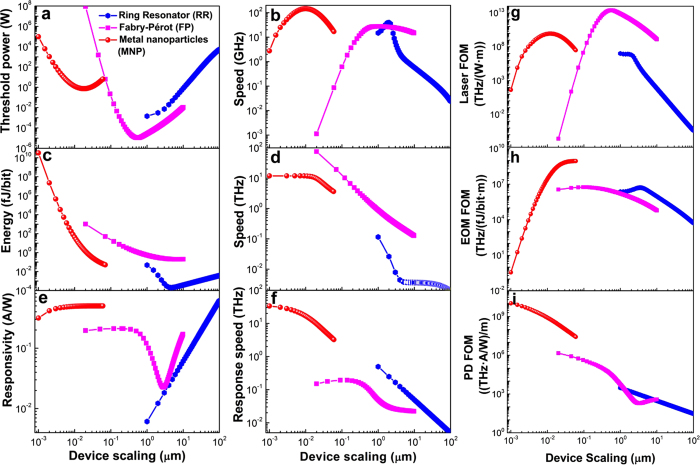
Scaling performance of electro-optic devices. (**a**) Laser electrical power threshold, *α*_*g*_ = 4700 cm^−1^ for gain medium of InGaAsP^20^, *τ*_*spo*_ = 10 ns^20^, *ν*_*sa*_*InP*_ = 15000 cm/s, *η*_*i*_ = 0.8, *ρ*_*c*_ = 1.0 ×10^−8^ Ω cm^2^, and *R*_*sh*_ = 16.5 Ω/, (**b**) Laser modulation speed, (**c**) Energy efficiency of EOM, *r*_*EO*_ = 100 pm/V_bias_, (**d**) Modulation speed of the EOM, *R*_*s*_ = 500 Ω, *R*_*dr*_ = 500 Ω, (**e**) Photodetector responsivity, *Δn*_*c*_ = 1.0 × 10^14^/m^3^, *n*_*c*_ = 1.0 × 10^19^/m^3^, *μ*_*n*_ = 1400 cm^2^/Vs, *ν*_*sa*_*Si*_ = 10000 cm/s, *C*_*n*_ = 1.1 × 10^−30^ cm^6^/s, *η*_*i*_ = 0.8, *V*_*bias*_ = 2.0 V, evaluated at *BW* = 10 GHz, and (**f**) Response speed of photodetector. (**g–i**) Device FOMs for laser, modulator, and photodetector devices.

For a laser high wall-plug conversion efficiency, a low threshold power (*P*_*th*_), compact geometry, and potentially a fast response times for direct modulation is desired^23^,^24^,^25^,^26^,^27^. The electrical power threshold = *I*^2^ × *R* is derived from the rate equations, where *I* is the drive current, and *R* the device resistance. For our device scaling law analysis the threshold is a function of *Q*, *V*_*m*_, *F*_*p*_, and a geometrical ratio of the exposed surface area of the gain material to the cavity volume, *S*_*a*_/*V*_*a*_^20^. Here the discussion of the electrical power threshold comparison for each cavity-based laser is related to the corresponding cavity-physics shown in Fig. 2, which indicates that *F*_*p*_ relates to the ratio of *Q* divided by the effective modal volume, *V*_*m*_, of the cavity. As for the light emission *F*_*p*_ ‘summarizes’ the effect of the cavity. Since *Q* for the RR is always orders of magnitude higher than *F*_*p*_, and *S*_*a*_/*V*_*a*_ is independent of RR radius, the threshold scales inversely with the Purcell factor. However, since *Q* is mostly flat for the RR at larger radius, the threshold actually scales proportionally with the mode volume *V*_*m*_ (Fig. 3a). However, for very small radii the bending loss becomes dominant and *Q* drops faster than the volume, hence slowing down the threshold reduction with scaling. For the FP, we need to consider two cases; for large length cavities, *Q* is much larger than *F*_*p*_ and the threshold reduces with scaling similarly to that of the RR (Fig. 3a). However, while *Q* drops with scaling, *F*_*p*_ increases and reaches a maximum *F*_*p*−*max*_ around 1 μm length, followed by a rapid drop. This explains the observed minima in the electrical power threshold. Note, *S*_*a*_/*V*_*a*_ is a constant that does not depend on the FP cavity length, i.e.,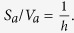 For the plasmonic cavity, *Q* for the MNP is orders of magnitude lower than *F*_*p*_, and *S*_*a*_/*V*_*a*_ = 3/*a* is inversely proportional to the radius of a metal nanoparticle. Thus, *Q* drops slower with scaling than *V*_*m*_ for particle radii corresponding the range above the maximum *F*_*p*_. The inverse trend for *Q* and *V*_*m*_ vs particle radius is true past *F*_*p*−*max*_, which explains the observed minimum. At the large radius limit, the MNP shows *F*_*p*_ ~ *Q* but shifts to *Q* ≪ *F*_*p*_ with scaling, and the threshold tracks first 1/*F*_*p*_ and then 1/*Q*. The latter can be seen by a faster threshold increase compared to the *F*_*p*_ dropping (Fig. 3a). The underlying explanation of the influence of high optical confinement leading to both high *F*_*p*_ and *β* is an altered pump-efficiency enhancement mechanism^28^,^29^. Compared to the lowest threshold from all three devices (FP, at *l* = 0.6 μm, *P*_*th*_ = 10^−5^ W) further scaling into the deep sub-micro regime results in a significant increase in the threshold power.

Wall-plug efficiency (WPE) represents the energy conversion efficiency with which the system converts electrical power into optical power. It is defined as the ratio of the laser total optical output power to the input electrical power consumption^30^. Here, the WPE of the laser is obtained by solving the rate equations and the power output expression, i.e., 
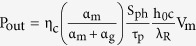
31, where η_c_ is the collection efficiency, α_g_ (α_m_) is the cavity absorption (mirror) losses per unit length, S_ph_ is the photon density, τ_p_ is the photon lifetime, h_0_ is the Planck constant, and c is the speed of light in free space. Thus, the WPE varies with the critical dimension. We find the maximum WPE to coincide with the same critical dimension where we noted the maximum Purcell factor. The highest WPEs are found for the FP laser approaching unity, while that of the ring and plasmon cavity are at 50% and 5%, respectively^20^. It is worth pointing out that the Schawlow Townes laser linewidth predicts a wider spectrum for nanolasers due to a high spontaneous emission factor. As such the use nanolasers for WDM applications is thus questionable, however, not discussed in the present work.

Relaxation oscillations are due to a periodic exchange of energy stored in laser gain medium by the population inversion and the cavity field intensity, showing a damped oscillation for a solitary laser. Semiconductor lasers normally exhibit strongly damped relaxation oscillations with high frequencies (e.g., GHz). The 3 dB bandwidth response for a direct-modulated laser is thus dominated by the relaxation oscillations of the gain medium especially at low pump rates. At high pump rates saturation occurs due to cavity damping^32^. In practice, we can estimate the laser speed by the small signal modulation near threshold of the spectral response function, which scales with a *β*-modified Purcell factor^20^,^33^. Therefore, all three cavity responses exhibit a maximum modulation bandwidth close to those lengths corresponding to maximum *F*_*p*_ (Fig. 3b); the ring improves rapidly with scaling, since *Q* drops and the cavity is not photon lifetime limited any more. FP tracks *F*_*p*_ well, but drops past 1 μm, due to the relatively low *Q* with scaling; *Q* continues to drop single exponentially, but *V*_*m*_ increases past the diffraction limit, due to the scaling of the optical confinement factor (*Γ*) with length^20^. The MNP-based laser is the only device able to surpass gain compression limited bandwidths (~10’s of GHz), and reach <100 GHz’s^32^,^34^. This is possible due to both the short photon lifetime inside the cavity, and simultaneously high *β*-factor. Regarding the laser threshold power, the MNP based laser has significantly higher thresholds than other cavities, and therefore by using external modulation strategy which modulates the continuous wave (CW) with an external modulator, the MNP based laser will consume more energy than over cavity based lasers which may neutralized the energy benefit of the MNP based modulator and detectors. However, together with its 10^4^ times smaller cavity footprint, such MNP lasers may bypass external electro-optic modulators, since the power overhead for cavity tuning and modulation can be saved, thus reducing optical link complexity and increasing link performance^35^. In addition, a more detailed external and internal modulation strategies analysis could be found in our previous work where we analyzed the link performance of all electronic, photonic, plasmonic and hybridization interconnects in the latency, energy, throughput and other comprehensive FOMs^36^.

## Modulator

An electro-optic modulator’s energy efficiency is determined by both the energy to charge the device’s electrical capacitor and the optical power penalty^37^,^38^,^39^,^40^,^41^,^42^. For the former, the critical field to obtain switching is proportional to the factor (*F*_*p*_*Q*)^−1^ 20. For a low-power device, the volume needs to reduce faster than the *Q* reduction upon scaling. The ring’s power shrinks with scaling to a corresponding *F*_*p−max*_ due to a flat *Q* and linear volume reduction. Past this point, the RR’s bending loss impacts the power scaling negatively leading to a dip approaching few photon (<1 aJ/bit) efficiencies. Unlike RR, the energy efficiency for the FP and MNP increases monotonically with scaling due to the 1/*F*_*p*_*Q* dominance (Fig. 3c). If footprint is a critical requirement, 10’s of nm small MNP devices with 100 aJ/bit are realizable. We note that the FP’s energy performance is limited to operating ranges above 1 fJ/bit mainly due to moderate *Q* and relatively high *V*_*m*_. The modulation bandwidth of the EOM is related to both the photon lifetime and *RC*-delay (i.e. *f*_*ph*_ and *f*_*RC*_), where the former is inversely proportional to *Q*, and the latter scales inversely with the critical length by means of electrical capacitance^20^,^43^. The RR is limited to modulation speeds of ten’s of GHz by both the long photon lifetimes and sizable electrical capacitances originating from diffraction limited large mode sizes (Fig. 3d). At the other extreme is the plasmonic-based modulator, which, in principle, can enable sub picosecond short response time given its small electrical capacitance and lossy cavity. Here we used the Pockels as an example modulation mechanism, since modulation speed exceeding 100 GHz have been experimentally demonstrated^44^. Similar conclusions hold for carrier-based modulation mechanisms as well, yet the material mobility may introduce modulation limits. Besides, the spherical shape of the MNP cavity is impractical to electrically contact the device, and even if realized, this might alter the optical mode^17^. The FP EOM connects the two regimes of high (RR) and low *Q* (MNP) allowing for high modulation speed due to a relatively lossy and compact cavity. While picosecond-fast EOMs are in principle perceivable^38^,^45^, state-of-the-art device drivers do not allow for such speeds, and future research should investigate co-design and co-integration of triggering techniques alongside novel devices^46^.

## Detector

Photodetectors contribute to defining bounds for both optical power limits and bit-error-rates (BER)^47^. Two crucial performance parameters for a detector are the responsivity and the bandwidth. Estimating a lower bound for the fundamental conversion efficiency, the minimum optical power (i.e. photo current times generated voltage) must exceed the noise floor to ensure sufficient signal quality and bit-error-rate. Applying shot-noise limits to the photocurrent and thermal noise to the voltage results in a minimum detector power equal to ~*k*_*B*_*T* times bandwidth (*k*_*B*_ = Boltzmann constant, *T* = temperature). This condition is, in reality, incorrect due to the statistical arrival of photons at the detector. Here, we consider a classical photon distribution along with non-radiative recombination effects such as defect and Auger-related recombination, which decrease the internal quantum efficiency and reduce the photocurrent output^20^. The governing equation for the responsivity denotes that a high value is achieved for short device sizes. Moreover, a high quality material such as withstanding high electric fields (i.e., *V*/*l*), a high mobility, and a low surface recombination velocity are also important to reach high performance^20^. For the interest of scaling laws the responsivity is inversely proportional to the external quantum efficiency (EQE), which depends on the absorption potential of the detector and is a function of the device length. The plasmonic MNP detector delivers a flat performance upon scaling, up to 0.5 A/W (Fig. 3e). The weak dependence arises from vanishing absorption- and scattering cross-sections in the short length limit; that is the absorption cross-section scales linearly with particle size. Similarly to other devices, the RR allows for higher responsivity when losses are low approaching 0.7 A/W. However when the radiation loss increases with scaling, the responsivity drops below 0.01 A/W due to the trivial length scaling. The FP cavity exhibits a minimum near the diffraction limit, which is a tradeoff between efficient absorption (long length limit) and a high exciton carrier collection efficiency (short length limit). For scaled devices the mirror loss becomes a constant, thus saturating the responsivity. Overall the plasmonic mode is able to produce competitively-high responsivities at small footprints.

There are several factors that influence the response time of a detector, such as transit and diffusion time scales of photo-carriers inside and outside the depletion region, and the *RC* delay (PN-junction photodiodes assumed). The 3 dB bandwidth scales inversely with device volume and is proportional to the EQE^20^. For large radii the RR-based detector scales inversely with length due to the high *Q*. Upon scaling, the contribution of increased optical losses reduce EQE and the carrier transit time shortens, thus increasing the bandwidth. The FP detector approaches a constant loss value for the large-scale limit. Upon scaling, the influence of the length-to-absorption loss increases comparably to the total loss, thus boosting the EQE and hence the device bandwidth. Similar to the EOM and laser, the MNP-based detector is able to receive data at much higher rates compared to photonic counterparts due to arguments such as low *RC* delay and short photon lifetimes. The commonly used detector benchmark is given by the responsivity-bandwidth product per footprint in units of A-ps/W-um^2^ gives values of 10^−3^, 10^−1^ and 10^4^ for the RR, FP, and MNP, respectively, showing the potential for high performance of sub-diffraction limited photonic detectors.

## Device Level Comparison

Next we apply a device-type specific performance vs. cost metric for each of the aforementioned devices. Such metric combines the device’ response speed and function-specific performance vs. ‘cost’, which we here express as scalability since it relates to physical wafer real estate. Device dependent performances for the laser and EOM are the threshold power and energy efficiency, respectively, which should both be minimized, and hence appear in the denominator of the device-FOM. The inverse is the case for the detectors’ responsivity which is to be maximized. Given the scaling laws for the laser threshold and speed, the FOM pertains the maxima of both the speed and inverse threshold. Note, we converted the threshold current to an electrical power via a scaling-dependent resistivity model^20^. The photonic FOMs for the RR and FP are 2–3 orders of magnitude higher compared to the MNP mainly due to the high power from the quantum point contact resistance^48^ and lossy cavity (Fig. 3g). The RR’s FOM interestingly improves monotonically with scaling and does not reduce even near its geometric minimal radius. The FP does not perform well beyond 1 micrometer scales due to the high thresholds and limiting speeds. The FOM for EOMs is relatively flat, which can be understood from the inverse relationship between the speed (~1/length) and energy (1/*F*_*p*_*Q*) scaling (Fig. 3h). The MNP is an exception, which is dominated by a 1/*F*_*p*_*Q* scaling. Interestingly, the photodetector displays a monotonically increasing FOM with scaling across all cavities (Fig. 3i); while the RR’s responsivity (in nominator of FOM) actually declines with scaling, the FOM increases due to the dependency of FOM = constant × 1/length. The FP continues to increase performance from the micrometer range down into the sub 100 nm since both responsivity and speed improve with scaling. The MNP continues to improve the FOM due to the almost ideal responsivity and short *RC* relay time of a sub 100 nm small device. As such, scaling does clearly improve the FOM for the detector, but its effect on EOMs and lasers is not monotonic.

## All-Optical Non-linear Device

**Figure 4:**
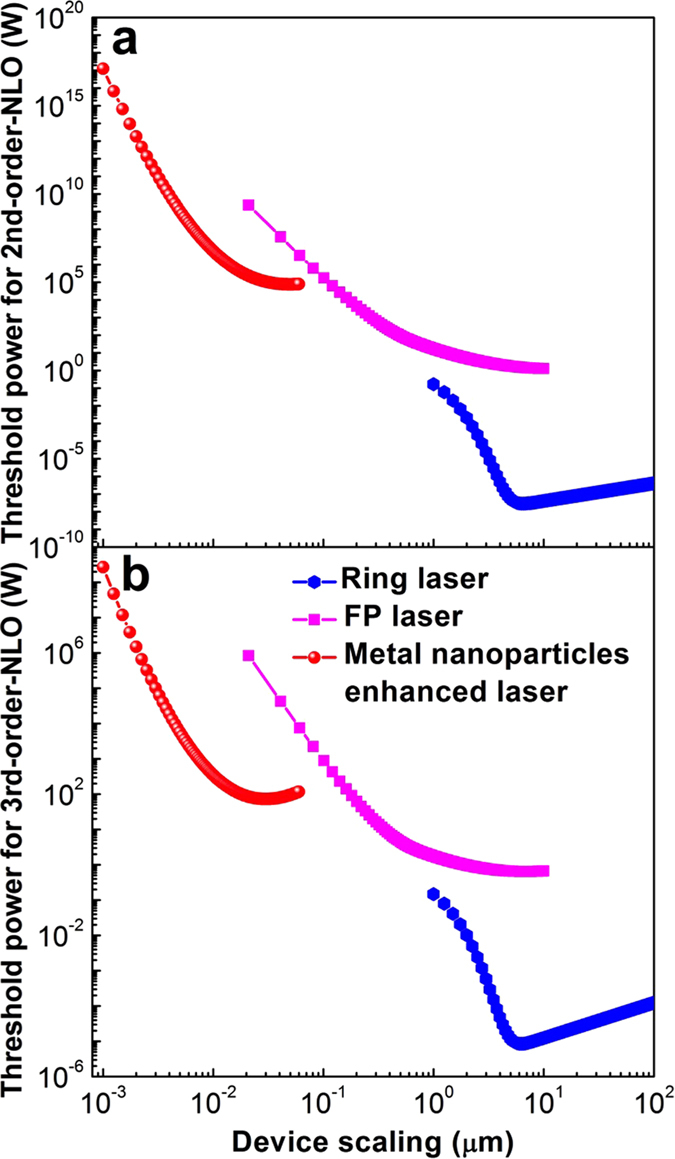
Scaling performance of all-optical switches based on. (**a**) Second-order and (**b**) third-order nonlinear effects. The parameters used for the simulations are *χ*^(2)^ = 2 × 10^−10^ m/V and *χ*^(3)^ = 5 × 10^−19^ m^2^/V^2^, respectively.

The three devices discussed so far are important for optical interconnects, where the computation is performed in the electronic domain. However, with growing interest in optical information technology, it is conceivable to perform computing as well using optics. Although, the exact functionality of optical computing is being debated to date, the key requirement is strong optical nonlinearity. The inherent material nonlinearity does not depend on carrier generation or physical motion of carriers. Hence it can be ultrafast, and the resulting nonlinear device does not suffer from electronic shot noise^49^,^50^. Unfortunately, this nonlinearity is generally rather weak, and requires LMI enhancement such as provided by a cavity. Thus, all-optical nonlinear devices provide an interesting test-case to elucidate the efficacy of scaling. For our analysis, we consider both the 2^nd^ and 3^rd^ order material nonlinearity, as they exhibit differing scaling performance with quality factors as reported recently^51^. We note that, in our analysis, we assume the scaling primarily affects the cavity, and not the nonlinear materials. Such assumption may not hold true if we rely on surface nonlinearity, as the effective nonlinearity might change as we scale. However, such surface nonlinearities are generally much weaker, and will not be suitable for building energy-efficient switches. To elucidate the scaling behavior, we analyze performance of an optical bistable system^50^,^52^. In such systems, the output power changes nonlinearly with the input power, with a sudden jump from one stable branch to another stable branch. We denote the input power where such sudden jump appears, to be the threshold power. This threshold power scales as 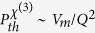 and 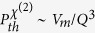
for 3^rd^ and 2^nd^ order nonlinear device, respectively. Clearly, a smaller mode-volume helps to reduce the optical power, but the quality factor plays a more significant role. This trend is evident from Fig. 4, where a lower *Q*-factor hurts the MNP and FP. The RR is a decent choice and the optimum nonlinearity can be achieved with ~5 μm radius. Of course, the actual value of the optimal radius depends on the material nonlinearity, but a large reduction in the radius cannot be realized. This indicates that it is unlikely that a digital optical computer will outperform a digital electronic computer due to extreme compactness of electronics. However, for different computing regimes, such as analog or neuromorphic computing, optics can still be competitive due to low loss in communication links as discussed next.

## Interconnect Link

**Figure 5:**
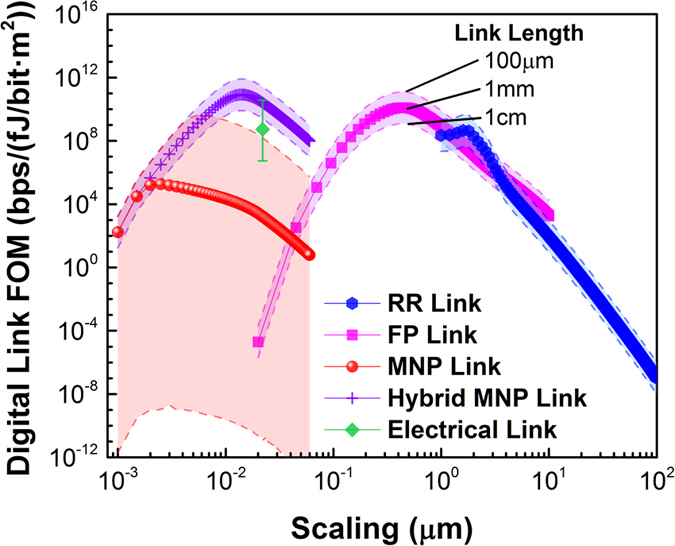
Scaling performance of a digital interconnect link technology for on-off key (OOK) modulation. Data points represent link lengths of 1 mm for chip-size signal communication, whereas dashed-lines denote how the link FOM changes for 100 μm (top) and 1 cm (bottom) communication distances. The electrical link (green) is based on the 22 nm technology node (similar link distances apply). The MNP chip length FOM is below unity (data clipped), due to the inherent losses in the link requiring high laser powers. Hybridizations^36^ (purple) where active components are plasmonic and passive data routing are photonics allow for highest FOMs for all length sales clearly outperforming electronics^20^. The RR, FP, and Hybrid MNP use Silicon photonics waveguides as interconnects (*α*_Si_ = 0.01 dB/mm), whereas the all-plasmonic MNP link deploys surface plasmon polariton waveguides (*α*_SPP_ = 44 dB/mm).

Based on the analysis of individual devices, we investigate scaling-based performance variations of various interconnect (IC) technology options^36^. We deploy the aforementioned FOM comprised of the link data speed divided by its ‘cost’ given by energy consumption times link footprint (Fig. 5). Here we compare photonic, plasmonic, electronic and hybrid IC types, where the three active devices (source, modulator and detector) are connected by different waveguide options as follows; the photonic ICs based-on active RR or FP devices are connected with conventional Silicon-on-Insulator (SOI) waveguides, whereas the active plasmonic MNP devices utilize surface plasmon metal waveguides. Further, we consider a photonic-plasmonic hybrid link, where all active devices are based on MNP cavities, but the passive interconnections are regular low-loss SOI waveguides (Fig. 5)^36^. As a comparison we relate these four photonic link FOMs to an electrical IC, which is based on the 22 nm technology node^53^.

The link FOM scaling model requires close attention to a variety of details that are different to the device level discussed above. For instance, the speed, i.e., link delay, is not simply the sum of all device delays, but determined by the slowest device on the entire link assuming bufferless data routing protocols, and the signal-to-noise (SNR) ratio of a noisy channel^54^. The latter depends on the bit error rate, where a BER = 10^−12^ was previously found to be appropriate for intra-chip communication^55^. Generally, the BER is a function of the variance of the noise model (i.e., Gaussian noise assumed), the detector’s responsivity, and the desired output current (*I*_*min*_) needed at the next stage in the circuit past the links photodetector^20^. *I*_*min*_ is naturally also a function of the link distance, which has to be provided for by the laser source. Here we explicitly show the link FOM not only as a function of device scaling, but also for three different IC link distances (0.1, 1 and 10 mm, dashed and solid lines Fig. 5). It is trivial to observe that longer links result in a lower FOM due to a higher laser power required to compensate the greater link loss. We note that the FOM is a complex combination of the various device’ FOMs; for instance, a higher device speed does not increase the link FOM linearly since the link delay is limited by the slowest device. The overall FOM follows that of the source, which is expected, since the source has to provide for the required signal quality required at the next stage in the circuit past the link. We show that the RR’s FOM improves significantly with scaling mainly due to the reduction in laser lower and higher data rates enabled by shorter cavity photon lifetimes. The FP and MNP links follow the device performance peaking just below 1 and 0.1 micrometers, respectively. The FP allows scaling by about 10 times compared to the RR with a small performance improvement, whereas the MNP enables 2 orders scaling past the highest FP FOM. A plasmonic-only link shows inferior FOMs compared to the RR and FP due to high source power and signal repetition every ten’s of micrometers. Altering the link length, introduces interesting changes in the FOM; the shorter the link, the higher is the FOM, since a lower source power is required, which is trivial. However, the photonic (RR and FP) links improve (worsen) with shorter (longer) link distance by about one order of magnitude, (shaded area, Fig. 5), whereas plasmonic links continue to improve (4–5 orders) for short link lengths, but become unusable for chip-scale lengths due to losses. With scaling the jitter-length sensitivity, a measure of FOM as a function of link distance, improves for all link types due to the laser power dominance over the link power, as indicative by the narrowing of the shaded area in Fig. 5. Therefore the difference of the propagation loss does not influence the link FOM significantly with scaling, except in the MNP case. The MNP exhibits the highest FOM for short links, which could be insightful for circuit designers. Furthermore, we show that the high jitter-length sensitivity of plasmonic links radically improves upon hybridization (purple data Fig. 5)^36^. Comparing the performance of optical to electronic link performance has relevance for photonic technology road-mapping; the inherent capacitive link performance limitations arising from charging electrical wires (green data, Fig. 5). As a result the electronic IC performance is actually surpassed by diffraction limited photonics, short distance plasmonics, or any-length hybrid photon-plasmonic links upon device scaling to near *F*_*p-max*_. This is consistent with the recent push of silicon and III-V photonics into low-*Q* rings with <5 μm ring radii^56^. In terms of scaling, the performance shows that plasmonics still improves its performance even past the electronic device size. Our analysis shows that plasmonics is only viable for either short links, or with hybridization.

## Conclusions

We have analyzed the performance of four relevant photonic devices, namely lasers, electro-optic modulators, photo-detectors, and all-optical switches with respect to their critical length scaling behavior based-on three different cavity types. We find that the interplay between cavity feedback and optical mode confinement strongly determines the device-inherent light-matter-interaction. Their respective device performance shows non-monotonic scaling behavior. In particular the interplay between spatial confinement of photons and cavity photon-storage, i.e., Purcell factor, determines to a large extend the device performance. While ring resonators enable low power lasers and modulators, their micrometer scale and long photon lifetimes limit their integration and device speed potential. Plasmonic metal particle-based cavities are the other extreme relying on small mode volumes and enable 10’s of nanometer small, high-speed devices, but are not as power efficient as microrings. All-optical non-linear devices are challenged by inherently high threshold values, and do not improve with scaling. Applying a speed-energy-footprint metric to compare interconnect performance of the various cavity-based technology options, we show that classical photonics outperforms electronics for short and long interconnect lengths, and plasmonic-based links offer even higher performance for signaling distances much shorter than typical chip die sizes. However, plasmonic links beyond 100 microns in length do not outperform photonics and suffer from high jitter-length-sensitivity. However, functionality separation among active (plasmonic) and passive (photonic) maintains highest link performance and allows competitive scaling compared to electronics, while maintaining long distance data delivery. These new insights can influence design choices at the circuit level and may pave the way for a photonic roadmap.

## Methods

Some details of this analysis are discussed in this method section, whereas further detailed elements appear in the supplementary online materials (i.e., ref. 20).

### Global Parameters

The following parameter conventions are applied: any length dependence that is not the scaling length is kept at the diffraction limit (λ/2*n*), unless otherwise stated. The operating wavelength is λ = 1550 nm, and *n* is the refractive index of the waveguide mode. For the laser analysis the surface recombination velocity, *ν*_*sa*_*InP*_, is a mid-range value of 1.5 × 10^4^ cm/s such as for a III-V material system^57^. For the electro optic modulator, micro-ring parameters: propagation loss *α*_*p*_ = 100 dB/cm, ε_r_ = 11.7 for Si, index *n* = 3.0, electrical series resistance *R*_*s*_ = 500 Ω, electrical device driver resistance *R*_*dr*_ = 500 Ω. For the Fabry-Perot parameters: mirror reflectivity: *R*_1_ = *R*_2_ = 95%. For the photodetector, the surface recombination velocity, *ν*_*sa*_*Si*_, is at a mid-range value of 1.0 × 10^4^ cm/s such as for Silicon^58^.

### Device Performance Analysis

For additional details on device and cavity analysis, symbol definitions, and methodology, refer to the supplementary online material^20^. The laser threshold current (Eqn. 1) under continuous pumping is derived from steady state rate equations considering surface recombination effects^28^. The electrical power threshold of a laser can be estimated by the total resistance (*R*_*t*_) times threshold current (*I*_*th*_) via 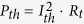. Here *I*_*th*_ can be determined by^28^,^34^


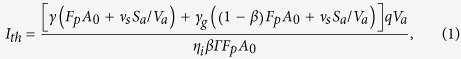


where *γ* is the total cavity mode loss rate per unit volume, which can be further expressed by *γ* = *γ*_*c*_ + *γ*_*g*_, *γ*_*c*_ is the loss rate due to cavity mirror loss and intrinsic loss, *γ*_*g*_ is the absorption rate per unit volume in the gain medium, and *γ*_*g*_ = (*α*_*g*_ · *c*/*n*), where *α*_*g*_ is the absorption coefficient per unit length of gain material. *A*_*o*_ is the natural spontaneous emission rate of the gain material, *v*_*s*_ is the surface recombination velocity, *S*_*a*_ and *V*_*a*_ are the exposed surface area (i.e., laser side walls and the gain material’s volume, respectively), *Γ* is the optical confinement factor quantifying the spatial overlap of the gain medium relative to a lasing mode, *η*_*i*_ is the current injection efficiency, and *q* is the electronic charge. *β* is the spontaneous emission coupling factor, denoting the fraction of the light of the lasing mode, relative to all available cavity modes. The cavity-based enhancement of the light-matter interaction is via *β*. The approximation for the *β* was first used for a nitride-based vertical microcavity surface-emitting laser^59^, where *β* is estimated by ~*F*_*p*_/(1 + *F*_*p*_). For a large *F*_*p*_, *β* approaches unity as expected. *R*_*t*_ is the total resistance of the laser, which is scaling dependent and can be evaluated by^60^


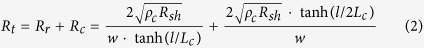


where *ρ*_*c*_ is the specific contact resistivity, *R*_*sh*_ is the sheet resistance of semiconductor materials under a metal contact, and *L*_*c*_ is the transfer contact length with 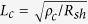.

The modulation bandwidth (Eqn. 3), i.e., 3-dB role-off speed, is estimated via the small signal response by observing the spectral response function^19^,


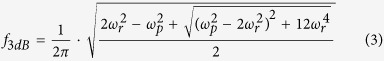


The oscillation relaxation frequency, *ω*_*r*_, of the laser is proportional to the cavity loss rate, *γ*, and is enhanced by *β*. The electro-optic modulator energy efficiency (Eqn. 4) is bounded by the electrical capacitive power consumption, the applied electric field, and cavity *Q-*factor^61^. The modulation speed (Eqn. 5) considers both the optical photon lifetime of the device cavity and the resistive-capacitive delay. Note, the latter includes both the device series and driver resistance^62^,^63^.


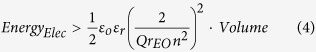



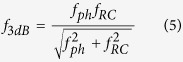


where *ε*_*o*_ is the vacuum permittivity, *ε*_*r*_ is the relative permittivity of the photonic material, *r*_*EO*_ is the Pockel’s coefficient of the EO material and *n* is the refractive index. 
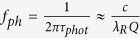
, 
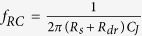
, where *R*_*s*_ is the modulator series resistance, *R*_*dr*_ is the driver impedance, and *C*_*J*_ is the modulator junction capacitance, here 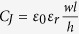.

The responsivity (Eqn. 6) of the photodetector quantifies the generated photo-current relative to the optical power input on the device^64^. Here non-radiative effects such as Auger and surface recombinations are considered. The photodetector response speed (Eqn. 7) is estimated from the small signal analysis^64^.


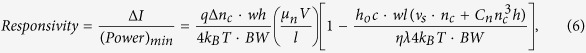



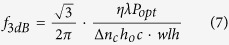


where Δ*I* is the photocurrent, Δ*n*_*c*_ is the excess carrier concentration, *μ*_*n*_ is the electrons mobility, *V* is the bias voltage, *h*_*o*_ is the Planck’s constant, *c* is the speed of light in free space, *n*_*c*_ is the generated carrier density, C_*n*_ is the Auger recombination coefficient of a material, *η* is the fraction of photons creating electron-hole pairs (i.e., quantum efficiency), *k*_*B*_ is the Boltzmann constant, *T* is the absolute temperature in kelvin, *BW* is the bandwidth of a photodector. *P*_*opt*_ is the optical power of the injected light.

We derive a separate FOM for each of the three electro-optic devices (i.e., laser, EOM and photodetector) using threshold power, energy efficiency, and responsivity, respectively, with their own speed and scaling (Eqns 8, 9, 10).


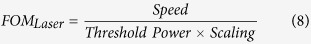



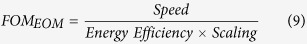



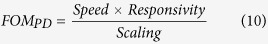


For the interconnect link analysis the FOM is defined as,


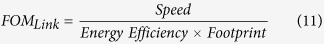


where the speed is no longer the device speed as we discussed in the previous sections, but can be considered as a link throughput for the entire channel, which can be predicted by the Shannon limit and noise level (see 3.1 SOM^20^).

## Additional Information

**How to cite this article**: Liu, K. *et al.* Fundamental Scaling Laws in Nanophotonics. *Sci. Rep.* **6**, 37419; doi: 10.1038/srep37419 (2016).

**Publisher’s note:** Springer Nature remains neutral with regard to jurisdictional claims in published maps and institutional affiliations.

## Supplementary Material

Supplementary Information

## Figures and Tables

**Figure 1:**
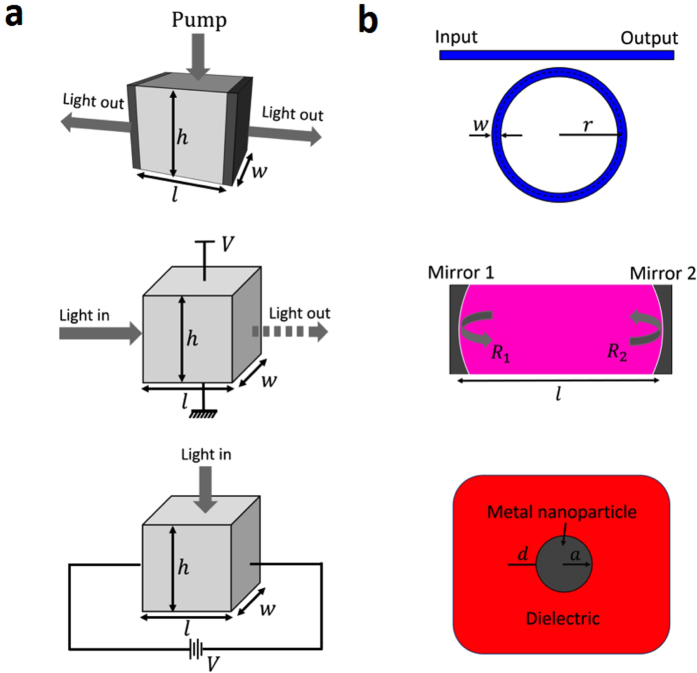
Schematic structures of devices and cavities. (**a**) Here we investigate performance scaling of four photonic devices, namely a laser source, an electro-optic modulator, a photodetector, and an all-optical nonlinearity-based switch (the latter is not shown). The physical device volume is given by the device geometry. (**b**) Here we utilize three device-underlying cavity types; namely a ring resonator (RR) cavity with the waveguide width, *w*, and the ring radius, *r*; a Fabry-Pérot (FP) cavity comprised of a dielectric material sandwiched by a pair of highly reflecting metal mirrors with the reflectivity of *R*_1_ and *R*_2_; and a plasmon cavity formed by metal nanoparticle (MNP) embedded in a dielectric, and *a* is the radius of metal nanoparticle. *d* represents the normal distance for the dipole position from the metal particle surface as is equal to 10 nm. The scaling parameters are *r*, for the RR, *l* for the FP, and *a* for the MNP cavity, respectively.
