# Supplementary material for: Fundamental Scaling Laws in Nanophotonics
Source: Sci Rep. 2016 Nov 21;6:37419. doi: 10.1038/srep37419 (PMC5116679; doi:10.1038/srep37419)
Supplement: Supplementary Information [file srep37419-s1.pdf]

Supplementary Online Information

Fundamental Scaling Laws in Nanophotonics

Ke Liu[1,4], Shuai Sun[1], Arka Majumdar[2,3], Volker J. Sorger[1*]

[1]Department of Electrical and Computer Engineering, The George Washington University, Washington, D.C. 20052, USA
[2]Department of Electrical Engineering, University of Washington, Seattle, WA 98195, USA
[3]Physics Department, University of Washington, Seattle, WA 98195, USA
[4]The Key Laboratory of Optoelectronics Technology, Ministry of Education, College of Electronic Information and Control Engineering, Beijing University of Technology, Beijing 100124, P.R. China

[*]Email: sorger@gwu.edu

Outline:

1. **Cavity Analysis**
   1.1 Ring Resonator
   1.2 Fabry-Pérot Cavity
   1.3 Metal Nanoparticle Plasmon Cavity
2. **Device Analysis**
   2.1 Laser Threshold Power
   2.2 Laser Modulation Speed
   2.3 Electro-optic Modulator Energy Efficiency
   2.4 Electro-optic Modulator Speed
   2.5 Photodetector Responsivity
   2.6 Photodetector Response Speed
3. **Link Analysis**
   3.1 Link Operating Speed
   3.2 Link Energy Consumption
   3.3 Link Scaling and Footprint

---

1. Cavity Analysis

In this study, we consider three types of resonators; namely a microring (ring), a Fabry-Pérot (FP), and a plasmonic metal nanoparticle (plasmon particle, Fig.1s). First we analyze each cavity type with respect to its cavity quality (Q)-factor, optical mode volume (V_m), and resulting Purcell factor (F_p). A formula widely used for the evaluation of F_p is given by

$$F_p = \frac{3}{4\pi^2}\left(\frac{\lambda_R}{n}\right)^3\left(\frac{Q}{V_m}\right), \qquad (1)$$

where Q is the cavity quality factor, V_m is the effective volume of electromagnetic energy of a resonant mode, λ_R is the resonant wavelength of the cavity, and n is the cavity material refractive index.

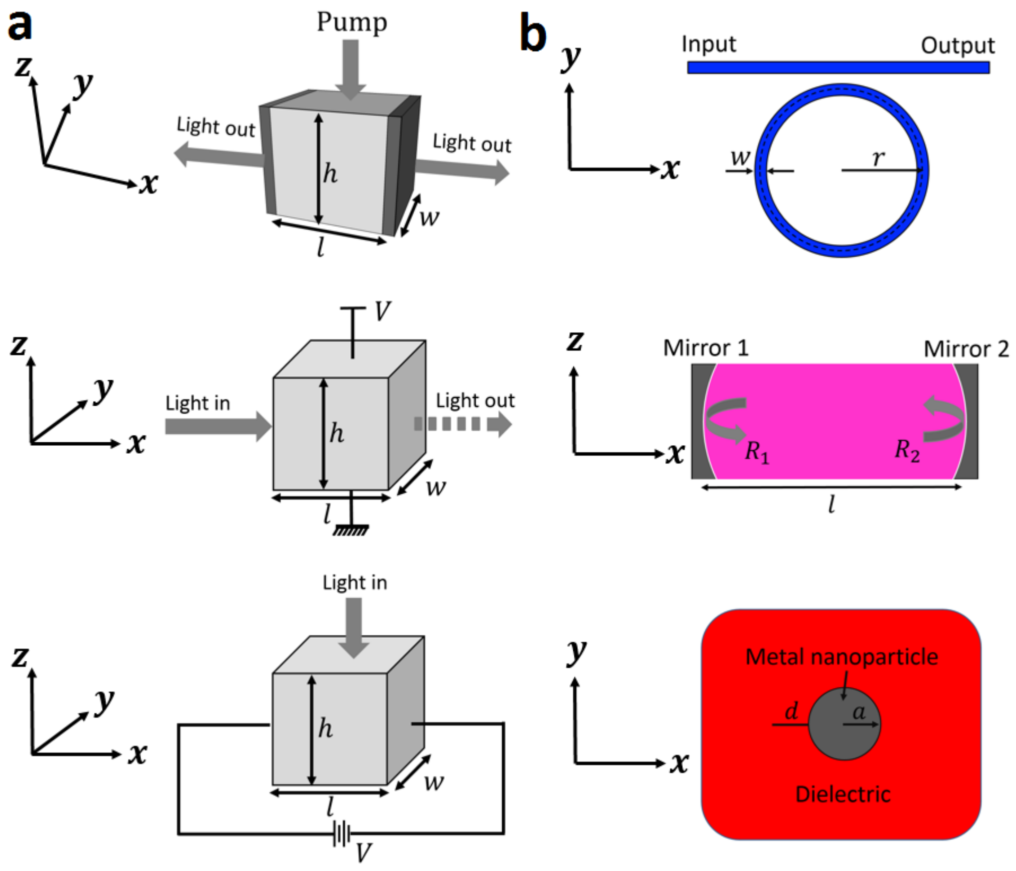

Figure 1s │ Schematic structures of devices and cavities. (**a**) A Fabry-Pérot laser, an electro-optic modulator, and a photodetector. w, l, and h are the device's width, length, and height, respectively. (**b**) We utilize three device-underlying cavity types; namely i) a ring resonator (RR) cavity with the waveguide width, w, and the ring radius, r, ii) a Fabry-Pérot (FP) cavity, showing a dielectric material sandwiched by a pair of highly reflecting mirrors with the reflectivity of R_1 and R_2 on the facets, and iii) a plasmon cavity formed by metal nanoparticle (MNP) embedded in a dielectric, and a is the radius of metal nanoparticle. d represents the normal distance for the dipole position from the metal particle surface as is equal to 10 nm in this study. The scaling parameters are r, for the RR, l for the FP, and a for the MNP cavity, respectively.

1.1 Ring Resonator

Here we investigate a ring resonator based-on the Silicon-on-insulator (SOI) platform (Fig.1sb). We derive an analytical expression of F_p related to physical parameters of a

ring resonator such as radius, loss coefficient, and waveguide cross-section, using coupled mode theory. The steady state transmission function at the critical coupling condition (i.e., loss in the ring equals to the fraction of light transmitted through the bus waveguide) is $\frac{P_{out}}{P_{in}} = \frac{2\mu^2+2\mu^2 cos(\theta)}{1+\mu^4+2\mu^2 cos(\theta)}$ [1], where μ is the loss in the ring, θ is the phase for resonance condition. At resonance, $\theta = (2m+1)\pi$, making $\cos(\theta) = -1$ and $P_{out} = 0$. When the resonator is completely off-resonant, then $\theta = 2m\pi$, making $\cos(\theta) = 1$. This leads to $\cos(\theta) = \frac{2\mu^2}{1+\mu^4}$. To convert the angle to a wavelength in full width at half maximum (FWHM), θ close to $= (2m+1)\pi$ is assumed at the FWHM wavelength point. We further express $\theta = (2m+1)\pi - \theta' = \frac{2\pi nL}{\lambda} = \frac{2\pi nL}{\lambda_o+\Delta\lambda} = \frac{2\pi nL}{\lambda_o}\left(1+\frac{\Delta\lambda}{\lambda_o}\right)^{-1} \approx \frac{2\pi nL}{\lambda_o} - \frac{2\pi nL\Delta\lambda}{{\lambda_o}^2}$, thus we have $\theta' \approx \frac{2\pi nL\Delta\lambda}{{\lambda_o}^2}$. This results in $\Delta\lambda = \frac{{\lambda_o}^2}{2\pi nL}\cdot\frac{1-\mu^4}{1+\mu^4}$. The round-trip loss depends on the length L, i.e., perimeter of a ring, then we can write $\mu = 10^{-\alpha L/20} = e^{-\frac{\alpha L}{20log_{10}(e)}}$. The Q factor is defined by $Q = \lambda/\Delta\lambda$, which is further given by

$$Q = \frac{2\pi Ln}{\lambda_R}\cdot\frac{1+e^{-k'L}}{1-e^{-k'L}} \tag{2}$$

Here the mode volume can be estimated by $V_m \approx Ltb$, where L is the perimeter of a ring, b denotes the lateral extent of electric field in the ring, which is related to the waveguide width, w, t is the waveguide thickness. Using Eqn. 1, F_p can thus be written by

$$F_p = \frac{3}{2\pi\cdot tb}\cdot\left(\frac{\lambda_R}{n}\right)^2\cdot\frac{1+e^{-k'L}}{1-e^{-k'L}} \tag{3}$$

where k' is a constant independent of the length given by $k' = \frac{\alpha}{5log_{10}e}$, and α is the total loss coefficient including propagation loss, α_p, and bending loss, α_{bend}. The bending loss becomes a major issue in the design of ring resonators with a small radius. Using the effective index method to a strip type waveguide, the resulting analysis leads to bending loss in dB's for $\Delta\theta$ of radius r as [2]

$$Loss_{bend} = -10\log[\exp(-\alpha_{bend}\Delta\theta r)], \tag{4}$$

where $\Delta\theta$ is a given angular section in radians, α_{bend} is the optical bending loss coefficient and is expressed by

$$\alpha_{bend} = \frac{\alpha_y^2}{k_o^3 n_e(1+\alpha_y w/2)}\frac{k_y^2}{(n_{e2}^2-n_{e1}^2)}\exp(\alpha_y w)\exp\left(\frac{-2\alpha_y^3}{3n_e^2 k_o^2}r\right), \tag{5}$$

where $k_o = 2\pi/\lambda_R$, $\alpha_y = k_o\sqrt{n_e^2-n_{e1}^2}$, and $k_y = k_o\sqrt{n_{e2}^2-n_e^2}$. $n_{e1}, n_e,$ and n_{e2} are the effective refractive indices for the 3-D waveguide structure divided into three slab waveguides (i.e., 2-D case), respectively.

The upper limit of Purcell factor in the microring resonator significantly depends on the radius and the total loss coefficient (main text, Fig. 2). For instance, the maximum F_p of ~2000 is achieved at a radius of ~7.0 μm with the condition of scattering loss coefficient of 1.0 dB/cm.

1.2 Fabry-Pérot Cavity

Fabry- Pérot cavity is the most common cavity structure, which consists of two highly reflecting mirrors on both sides forming standing light wave oscillations (Fig. 1sb). Here we can derive its Purcell factor related to the length of the FP cavity. The energy in the cavity decays due to internal losses and mirror transmissions. After one round trip, the intensity, $I(t_1)$, becomes

$$I(t_1) = R_1 R_2 (1 - T_{loss})^2 I_{o.} \quad (6)$$

where R_1 and R_2 are the optical reflectivity on the cavity facets, T_{loss} is the fractional internal loss per pass, which considers the summation of light absorption in the cavity, i.e., $\alpha_{abs\_total} = 1 - e^{-\alpha_{abs} l}$, and field penetration loss in the mirrors, $\alpha_{pen} = (\frac{4\pi\kappa_M}{\lambda_R})\delta_s \cdot (\frac{2\delta_s}{2\delta_s + l})$, where α_{abs} is the absorption coefficient, and $\alpha_{abs} = 4\pi\kappa_D/\lambda_R$, κ_D and κ_M are the extinction coefficients of dielectric and metal materials, respectively, δ_s is the penetration depth at which the field magnitude drops to $1/e$ of the surface value, and is given by $\delta_s = \sqrt{\frac{\lambda_R \varepsilon_o c}{\pi\sigma}}$ [3], σ is the electrical conductivity in mho/m, and ε_o is the vacuum permittivity. After m round trips at time $t_m = 2ml \cdot n/c$, where c is the light speed in free space, the intensity and the total number of photons, $\phi(t_m)$, can be written by,

$$I(t_m) = [R_1 R_2 (1 - T_{loss})^2]^m I_{o,} \quad (7)$$

$$\phi(t_m) = [R_1 R_2 (1 - T_{loss})^2]^m \phi_{o,} \quad (8)$$

respectively, where ϕ_o is the number of photons initially present in the cavity. Equation (7) can be further set as an exponential decay, $\phi(t_m) = [\exp(-t/\tau_{phot})]\phi_{o,}$ τ_{phot} is the photon lifetime. Therefore, τ_{phot} of a FP cavity can be derived by

$$\tau_{phot} = -\frac{2nl}{c \cdot log[R_1 R_2 (1 - T_{loss})^2]} \quad (9)$$

Quality factor is proportional to τ_{phot} by $Q = 2\pi c\tau_{phot}/\lambda$, and Q can thus be achieved. Toward evaluating F_p of the FP cavity, V_m is estimated by the product of an effective mode area and the cavity length, i.e., $V_m = A_{eff} \cdot l = \frac{A_a}{\Gamma_a(l)} l = \left(\frac{\lambda_R}{2n}\right)^2 \frac{l}{\Gamma_a(l)}$ [4], where A_a is the cross-section area of the FP cavity, and $\Gamma_a(l)$ is the mode confinement factor depending on the cavity length. Borrowing the concept of slab waveguide mode confinement, $\Gamma(l) = [1 + (\frac{2}{\alpha_e l}) \frac{cos^2(\frac{kl}{2})}{1 + \sin(kl)/(kl)}]^{-1}$[5] is used here to evaluate the FP cavity mode confinement, where k is the wave vector, and $\alpha_e = k \cdot tan(kl/2)$ based on an eigenequation.

Using Eqn. 1, the analytical expression of F_p for the FP cavity can be derived. Similar to the ring case, we find a maximum F_p around a length of 0.5 μm for the FP

cavity. Compared to the ring cavity, the maximum F_p for the FP cavity is reached at footprint that is over 1200 times smaller (main text, Fig. 2). However, the overall F_p is significantly lower (1.5%) than the ring case. This is mainly due to the lower Q factor, which originates from optical field penetrating the metal mirror. In addition, as the length of FP cavity is further down scaled, e.g, <180 nm, the FP cavity is changed from being "FP" to being a "metal-insulator-metal" (MIM) structure in such a short length limit. We numerically validate this modal configuration change using Lumerical FDTD and Mode solutions software, respectively (Fig. 2s). As the length of >180 nm for the FP cavity, we can observe a longitudinal mode along the cavity. However, as the length of <180 nm, only a plasmonic mode can be found in the FP cavity (i.e., MIM).

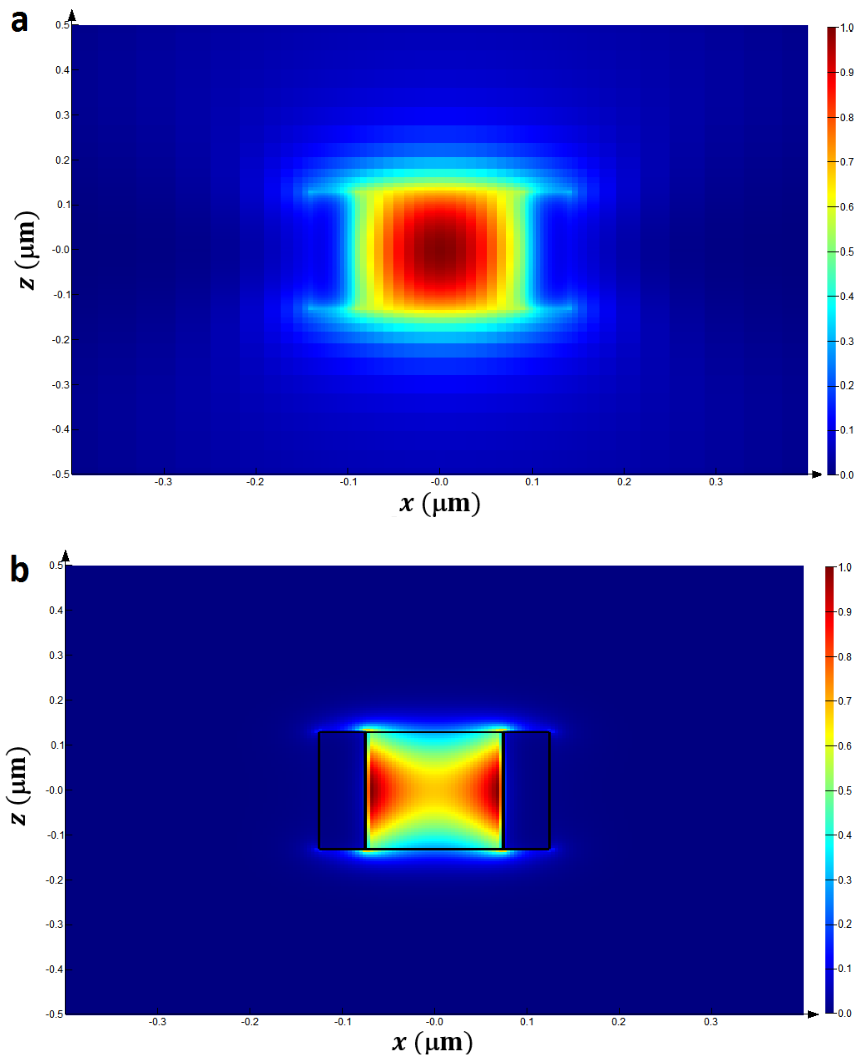

Figure 2s | Comparison of electric field confined in the FP cavity. (**a**) A longitudinal mode along the cavity with a length of 180 nm. (**b**) A plasmonic mode in the MIM structure with the FP cavity length reduced up to 150 nm.

1.3 Metal Nanoparticles Plasmon Cavity (MNP)

Plasmonic cavities can overcome the diffraction limit of light, thus enabling nanophotonic devices with nanoscale size, such as mode dimensions below the wavelength of the light. Here we introduce a plasmon cavity formed by metal nanoparticles embedded in a dielectric material to study the scaling of Purcell factor (Fig.1sb). Such a configuration interacts relatively strongly with electromagnetic radiation of wavelengths that are far larger than the particle due to the nature of the dielectric-metal interface between the medium and the particles, exhibiting non-classical scattering, absorbance, and coupling properties based on their geometries and relative positions. Purcell factors of this MNP cavity can be modeled as a ratio of the effective density of the SP modes, ρ_{SP}, relative to that of the radiation continuum, ρ_{rad}, [6],

$$F_p = \frac{\rho_{SP}}{\rho_{rad}} = \left[\frac{L(\omega_o)}{V_m}\left(\frac{r}{r+d}\right)^6\right]\left[\frac{1}{3\pi^2}\left(\frac{2\pi}{\lambda_R}\right)^3\frac{1}{\omega_o}\right]^{-1} \tag{10}$$

where $L(\omega_o)$ is the standard Lorentzian shape that can be simplified by $L(\omega_o) = 2/(\pi\gamma_d)$ at the SP resonance frequency, γ_d is the dipole decay rate. $V_m = \frac{4}{3}\pi a^3\left(1 + \frac{1}{2\varepsilon_D}\right)$ [7], where ε_D is the dielectric constant of the surrounding media, and a is the radius of metal nanoparticle. This Purcell factor exhibits a strong dependence on the metal particle size, showing that F_p increases initially with the particle size and then decreases as the particle volume increases (main text, Fig. 2). Here d =10 nm is used in this work. Note, we can derive the Q factor expression from Eqn. 1 based on Eqn. 10 for device applications.

2. Device Analysis

2.1 Laser Electrical Power Threshold

Here we discuss how we achieve Fig. 3a in the main text. For the laser electrical power threshold, the condition $P_{th} = I_{th}^2 \times R_t$ is used, where I_{th} is the laser threshold current that can be derived from the steady state rate equations, and R_t is the total resistance. First, we introduce the steady state rate equations for laser devices.

As considering surface recombination effects, the steady state rate equations under continuous pumping are expressed by [8],

$$\frac{dN}{dt} = P_{pump} - AN - \beta\Gamma AS(N - N_{T0}) - (\nu_s S_a/V_a)N \tag{11}$$

$$\frac{dS}{dt} = \beta AN + \beta\Gamma AS(N - N_{T0}) - \gamma S \tag{12}$$

where P_{pump} is the pump rate, and $P_{pump} = \eta_i \frac{I}{qV_a}$, I is the injection current, η_i is the current injection efficiency, V_a is the active gain volume (i.e., $V_a = wlh$), q is the electronic charge. S is the photon number of a single lasing mode, N is the excited state

population density, A is the spontaneous emission rate, which can be modified by the Purcell effect via $A = F_p A_o$, where A_o is the natural spontaneous emission rate of the material, and $A_o = 1/\tau_{sp0}$, τ_{sp0} is the spontaneous emission lifetime of gain medium. Γ quantifies the overlap between the spatial distribution of gain medium relative to a lasing mode, β is the spontaneous emission coupling factor that can be estimated by $\sim F_p/(1+F_p)$, S_a is the exposed surface area (i.e., side walls of the device), ν_s is the surface recombination velocity, N_{T0} is the excited state population at transparency ($\sim 10^{18}$ /cm^3), γ is the total cavity mode loss rate per unit volume, which can be further expressed by $\gamma = \gamma_c + \gamma_g$, γ_c is the loss rate per unit volume due to cavity mirror loss and intrinsic loss, can be evaluated by $\gamma_c = 2\pi c/(Q\lambda_R V_m)$; γ_g is the absorption rate per unit volume due to the gain medium absorption, and $\gamma_g = (\alpha_g \cdot c/n)/V_m$, where α_g is the absorption coefficient per unit length.

Secondly, we derive an analytical expression of I_{th} for the laser device. Solving Eqn. 11 and 12, N as a function of S is given by,

$$N = \frac{P_{pump} - \gamma S}{A(1-\beta) + \nu_s S_a/V_a}. \quad (13)$$

Substituting Eqn. 13 into Eqn. 11, and re-write the expression towards obtaining a quadratic equation with one unknown parameter, S, we obtain

$$\gamma S^2 - S\left[P_{pump} - \frac{\left(A + \frac{\nu_s S_a}{V_a}\right)\gamma + \gamma_g\left[(1-\beta)A + \frac{\nu_s S_a}{V_a}\right]}{\beta \Gamma \mathrm{A}}\right] - \frac{P_{pump}}{\Gamma} = 0. \quad (14)$$

Referring to Eqn. 2 in the ref. 5, the threshold pump rate, P_{pump_th}, can be achieved by

$$P_{pump\_th} = \frac{\left(A + \frac{\nu_s S_a}{V_a}\right)\gamma + \gamma_g\left[(1-\beta)A + \frac{\nu_s S_a}{V_a}\right]}{\beta \Gamma \mathrm{A}}. \quad (15)$$

Resulting in the threshold current thus given by

$$I_{th} = \frac{\left[\gamma\left(F_p A_0 + \nu_s S_a/V_a\right) + \gamma_g\left((1-\beta)F_p A_0 + \nu_s S_a/V_a\right)\right] q V_a}{\eta_i \beta \Gamma F_p A_0} \quad (16)$$

For our device scaling law analysis the threshold as a function of Q, V_m, F_p, and S_a/V_a is of interest since they depend on the critical cavity scaling length. Here, we are interested in simplifying and restating Eqn. 16 with its explicit dependency on Q, V_m, F_p, and S_a/V_a. Starting with the cavity loss rate, γ is inversely proportional to Q, and $\beta \cong F_p/(1+F_p)$ as stated before. Thus, based on table S1, Eqn. 16 simplifies to

$$I_{th} \propto \frac{\frac{F_p}{Q} + (1-\beta)F_p + \frac{S_a/V_a}{Q} + S_a/V_a}{\beta F_p} = \frac{1}{Q} + \frac{1 + S_a/V_a}{F_p} + \frac{1 + S_a/V_a}{F_p Q} + \frac{S_a/V_a}{F_p^2 Q} + \frac{S_a/V_a}{F_p^2} \quad (17)$$

Since Q for the RR is always orders of magnitude higher than F_p, and S_a/V_a is a constant that is independent on the RR radius, i.e., $S_a/V_a = \left(\frac{1}{t} + \frac{2}{w}\right)$, the threshold scales inversely with the Purcell factor. However, since Q is mostly flat for the larger RR, the threshold

actually scales proportionally with the mode volume V_m. However, for very small radii the bending loss becomes dominant and Q drops faster then the volume, hence slowing down the threshold reduction with scaling. Similar arguments apply to the FP laser threshold. However, for the MNP laser threshold, Q for the MNP is the orders of magnitude lower than F_p, and $S_a/V_a = 3/a$ is inversely proportional to the radius of a metal nanoparticle, the threshold scales inversely with the quality factor.

Table S1. Numerical values calculated for the terms of F_pA_0, ν_sS_a/V_a, and $(1-\beta)F_pA_0$ for three cavities, respectively. Three values are selected for each cavity, i.e., one at maximum F_p, the other with larger scaling, and the third one with lower scaling.

Parameters	Cavities	Numerical values
F_pA_0	RR	2.1×10^{11} /s @Fp_{max}
		1.3×10^{10} /s @Larger scaling
		3.8×10^{9} /s @ Lower scaling
	FP	2.8×10^{9} /s @Fp_{max}
		1.4×10^{9} /s @Larger scaling
		1.7×10^{6} /s @ Lower scaling
	MNP	2.4×10^{10}/s @Fp_{max}
		2.9×10^{9} /s @Larger scaling
		8.8×10^{8} /s @ Lower scaling
$\nu_s\, S_a/V_a$	RR	1.7×10^{9} /s @ Scaling of Fp_{max}
		1.7×10^{9} /s @Larger scaling
		1.7×10^{9} /s @ Lower scaling
	FP	1.7×10^{9} /s @ Scaling of Fp_{max}
		1.7×10^{9} /s @Larger scaling
		1.7×10^{9} /s @ Lower scaling
	MNP	4.5×10^{10}/s @ Scaling of Fp_{max}
		7.5×10^{9} /s @Larger scaling
		4.5×10^{11}/s @ Lower scaling
		1.0×10^{8} /s @Fp_{max}

$(1-\beta)F_pA_0$	RR	9.9×10^{7} /s @Larger scaling
		9.7×10^{7} /s @ Lower scaling
	FP	9.6×10^{7} /s @Fp_{max}
		9.3×10^{7} /s @Larger scaling
		1.7×10^{6} /s @ Lower scaling
	MNP	1.0×10^{8} /s @Fp_{max}
		9.7×10^{7} /s @Larger scaling
		9.0×10^{7} /s @ Lower scaling

Resistance at metal-semiconductor contacts is one of the major obstacles to decreasing power efficiency in laser devices. Thirdly, to study the resistance scaling, we borrow the derivation of contact resistance framework from metal-oxide-semiconductor field-effect transistor (MOSFET) device by using transmission line model [9]. The resistive load contribution to the series source (R_s) and drain (R_d) resistance is expressed by [9],

$$R_r = R_s + R_d = \frac{2\sqrt{\rho_c R_{sh}}}{w \cdot \tanh\left(l/L_c\right)} \quad (18)$$

where ρ_c is the specific contact resistivity, R_{sh} is the sheet resistance of semiconductor materials under a metal contact, and L_c is the transfer contact length with $L_c = \sqrt{\rho_c/R_{sh}}$. The corresponding contact resistance, can be given by [9],

$$R_c = \frac{2\sqrt{\rho_c R_{sh}} \cdot \tanh\left(l/2L_c\right)}{w} \quad (19)$$

The total resistance is the summation of the contact resistance and the resistive load, i.e., $R_t = R_c + R_r$, when assuming Eqn. 18 and 19 to apply for the laser driver (i.e., contact resistance due to source-drain voltage applied + resistive load). Note, for the metal nanoparticles plasmon laser, we use a resistive point contact, which indicates that $w = 2a$ and $l = 2\pi a$. Here we use a global ρ_c of 1.0×10^{-8} Ω cm^2 for heavily doped n-type InGaAs:Si layers with 6×10^{19} cm^3 and a sheet resistance of R_{sh} =16.5 Ω/□ for InGaAs [10]. To readily understand laser threshold equation, we summarize that how we achieve those parameters and the corresponding values used in Eqn.16, 18, and 19 (Table S2).

The Joule heating with a direct current injection leads to temperature increase due to the contact resistance and resistive load existing in the laser device, which can lead to increasing the laser threshold power. The two factors can influence P_{th}, i.e., I_{th} can be

decreased by a larger Q and F_p, while a wider contact width and a smaller ρ_c are beneficial to reducing R_t.

Table S2. Summary of parameters decomposition for laser threshold equations

Parameters	Values/equations	Note
γ_c	$2\pi c/(Q\lambda_R \cdot V_m)$	Q, V_m can be extracted from main text, Fig. 2a and b
$\boldsymbol{F_p}$	can be extracted from main text, Fig. 2c	
$\boldsymbol{\tau_{sp0}}$	10 ns	Spontaneous emission lifetime of InGaAsP [11]
$\boldsymbol{A_0}$	$1/\tau_{sp0}$	
v_{sa_InP}	15000 cm/s	Ref.11
β	$\sim F_p/(1+F_p)$	
A	$F_p A_0$	
$\boldsymbol{\alpha_g}$	4700 cm^{-1}	For a gain medium of InGaAsP [12]
γ_g	$(\alpha_g \cdot c/n)/V_m$	V_m can be extracted from main text, Fig. 2b
γ	$\gamma = \gamma_c + \gamma_g$	
Γ (FP)	$[1+(\frac{2}{\alpha_e l})\frac{cos^2(\frac{kl}{2})}{1+\sin(kl)/(kl)}]^{-1}$	Refer to the section of 1.2 Fabry-Pérot Cavity
Γ (RR and MNP)	0.8	
η_i	0.8	
ρ_c	1.0×10^{-8} Ω cm^2	Specific contract resistivity of InGaAs [10]
R_{sh}	16.5 Ω/□	Sheet resistance of InGaAs [10]
L_c	$\sqrt{\rho_c/R_{sh}}$	

Wall-plug efficiency (WPE) indicates the energy conversion efficiency for lasers, which is defined by $WPE = P_{out}/P_{th}$, where P_{out} is the optical output power, which is expressed by $P_{out} = \eta_c(\frac{\alpha_m}{\alpha_m+\alpha_g})\frac{S_{ph}}{\tau_p}\frac{h_0 c}{\lambda_R}V_m$, where η_c is the collection efficiency, α_g (α_m) is the cavity absorption (mirror) losses per unit length, S_{ph} is the photon density, and $S_{ph} = S/V_a$, τ_p is the photon lifetime, h_0 is the Planck constant, and c is the speed of light in free space. S can be obtained by solving the rate equations (i.e., Eqn. 11 and 12). Here we use $\alpha_m = \frac{1}{2(2\pi r)}\cdot\ln\left(\frac{1}{R_{F1}\cdot R_{F2}}\right)$, $\frac{1}{2l}\cdot\ln\left(\frac{1}{R_1\cdot R_2}\right)$, and $\frac{1}{2h}\cdot\ln\left(\frac{1}{R_{F1}\cdot R_{F2}}\right)$ for RR, FP, and MNP-cavity based lasers, respectively, where R_{F1} and R_{F2} are the Fresnel reflectivity at the waveguide facets. The electrical power threshold for each cavity based laser shown in Fig. 3a is known, the WPE can be calculated (Fig. 3s).

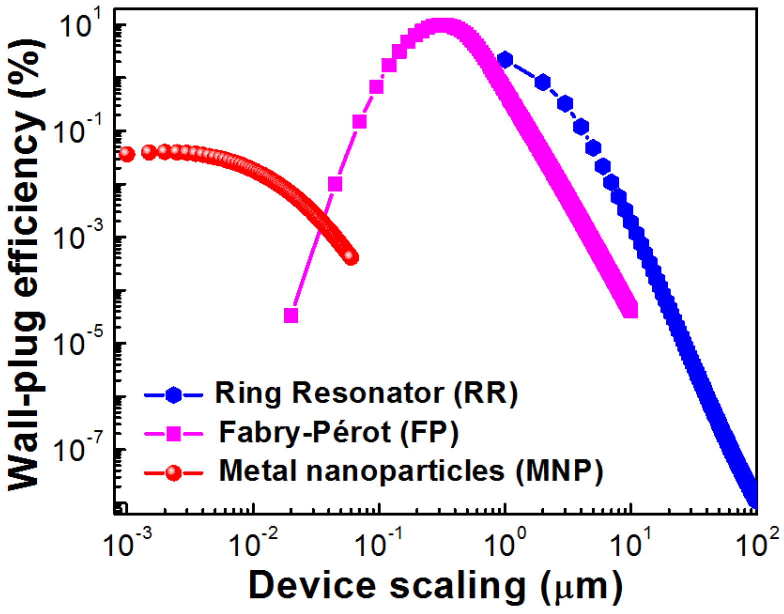

Figure 3s | Scaling performance of wall-plug efficiency for lasers.

2.2 Laser Modulation Speed

The modulation bandwidth (i.e., 3-dB role-off speed) can be estimated through the small signal response by observing the spectral response function [13],

$$H(\omega) = \frac{\beta\Gamma_T(1+S_0)}{\sqrt{(\omega^2-\omega_r^2)^2+\omega^2\omega_p^2}} \quad (20)$$

where ω is the optical cavity angular frequency, Γ_T is the transition rate of excited state population, which is equal to the spontaneous emission rate, A. $\omega_p = \gamma + \Gamma_T(1 - \beta N_0 + \beta S_0)$, and $\omega_r^2 = \Gamma_T[\gamma(1+\beta S_0) - \beta(1-\beta)\Gamma_T N_0]$, S_0 and N_0 are the steady-state photon number and population inversion number, respectively, and Γ_T=$F_p A_o$. The time response of a nanolaser is characterized by the modulation bandwidth, f_{3dB}, defined as the

frequency at which the response function decays to half of its zero-frequency value (i.e., $H(\omega)/H(0)$), which is expressed by

$$\frac{H(\omega)}{H(0)} = \frac{\omega_r^2}{\sqrt{\left(\omega^2-\omega_r^2\right)^2+\omega^2\omega_p^2}} \tag{21}$$

We re-write Eqn. 21 for the modulation bandwidth with $H(\omega)/H(0) = 1/2$,

$$f_{3dB} = \frac{1}{2\pi} \cdot \sqrt{\frac{2\omega_r^2-\omega_p^2+\sqrt{(\omega_p^2-2\omega_r^2)^2+12\omega_r^4}}{2}} \tag{22}$$

For the comparison of laser threshold power and modulation speed, three types of cavities including RR, FP, and MNP cavities are configured for the laser device. The corresponding energy expression of Eqn.16 and the bandwidth expression of Eqn. 22 can use each cavity's Q and F_p formula for the device scaling.

2.3 Electro-optic Modulator Energy Efficiency

High-speed EOMs need to be power efficient and ultra-compact. The energy efficiency of an EOM is given by the charging the device capacitor, ½ CV^2, where C is the device capacitance, and V is the driving voltage [14]. This expression takes the resistive energy loss of the modulator device itself into account during the charge-discharge cycles, ignores the power consumed by the driver circuit, and hence provides a lower bound for the switching energy [15]. For field-effect devices the refractive index change is governed by an electric field, E, where $E = V/h$, h is the thickness of a device volume. The device capacitance is calculated by $C = \varepsilon_o\varepsilon_r wl/h$. The electrical energy efficiency, $Energy_{Elec}$, can be further expressed by

$$Energy_{Elec} = \frac{1}{2}\varepsilon_o\varepsilon_r \cdot E^2 \cdot whl = \frac{1}{2}\varepsilon_o\varepsilon_r \cdot E^2 \cdot (Volume). \tag{23}$$

The index change for an EOM with Pockel's effect is related to electric field by $\Delta n_p = \frac{1}{2} r_{EO} n^3 E$, r_{EO} is the linear electro-optic coefficient (i.e., Pockels coefficient) of the cavity material. The resonant wavelength shift of the cavity $\Delta\lambda$ exhibits an fast response to E by $\Delta\lambda = \frac{\Delta n_{eff}}{n_g} \cdot \lambda_R \approx \frac{1}{2} r_{EO} n^2 \lambda_R E$ through taking the group index n_g to be equal to the effective index [15]. The photon lifetime is related to Q by $\tau_{phot} = \frac{Q \cdot \lambda_R}{2\pi c}$, and Q is defined by $Q = \lambda_R/\delta\lambda$. Assuming the cavity linewidth approximated to its bandwidth, BW, Q factor can be further expressed by $Q \approx \frac{2\pi c}{\lambda_R \cdot BW}$. We note that BW is the modulation speed in Hz. For a cavity based EOM, the condition of $\Delta\lambda > \delta\lambda$ was taking into account. We obtain the electric field,

$$E > \frac{\lambda_R \cdot BW}{\pi r_{EO} n^2 c}. \tag{24}$$

The electrical energy efficiency can thus be bounded by a quality factor,

$$\begin{aligned} Energy_{Elect.} &= \tfrac{1}{2}CV_{bias}^2 \\ &= \tfrac{1}{2}\left(\varepsilon\frac{WL}{h}\right)(h)^2 E_{critical}^2 \\ &= \tfrac{1}{2}\varepsilon\cdot E_{critical}^2\cdot(WLh) \\ &= \tfrac{1}{2}\varepsilon\cdot E_{critical}^2\cdot(Volume) \\ &> \tfrac{1}{2}\varepsilon\cdot\left(\frac{2}{r_{EO}n^2Q}\right)^2(Volume) \\ &\propto \frac{1}{F_p\cdot Q} \end{aligned} \tag{25}$$

2.4 Electro-optic Modulator Speed

The overall modulation bandwidth of an EOM is related to the photon lifetime and RC-limited bandwidth (i.e., f_{ph} and f_{RC}) through this expression [16],

$$f_{3dB} = \frac{f_{ph}f_{RC}}{\sqrt{f_{ph}^2+f_{RC}^2}} \tag{26}$$

where $f_{ph} = \frac{1}{2\pi\tau_{phot}} \approx \frac{c}{\lambda_R Q}$, $f_{RC} = \frac{1}{2\pi(R_S+R_{dr})C_J}$, R_S is the modulator series resistance, R_{dr} is the driver impedance, and C_J is the modulator junction capacitance, here $C_J = \varepsilon_0\varepsilon_r\frac{wl}{h}$. Eqn. 26 indicates that the modulation bandwidth is limited by the Q factor.

For the comparison of EOM energy efficiency and modulation speed, we configure an EOM with cavity enhanced by RR, FP, and MNP cavity, respectively. The corresponding energy expression of Eqn. 25 and bandwidth expression of Eqn. 26 only use each cavity's Q formula for the device scaling.

2.5 Photodetector Responsivity

Photodetectors are key components in photonic integrated circuits due to their crucial contributions to optical power limits, and bit-error-rates. The minimum power received can be considered as the product of $Current(I)$ and $Voltage$ (V), which must be above the $Noise$ limit (Fig. 1sa). Two fundamental effects that lead to signal degradation are thermal noise and shot noise. In terms of thermal noise, assuming a single electron charge at room temperature, $qV > energy@300\,K \cong 2\,k_BT$, which cannot detect mid-IR wavelength range unless cooling the system is applied. The shot noise can be evaluated by $(\Delta I)^2 = 2qI\times BW$ at a single-noise-ratio of $\sqrt{I/(2q\times BW)}$, leading to $I > 2q\times BW$. The minimum power, P_{opt}, is thus calculated via $IV \geq 2q\cdot BW\times\frac{2k_BT}{q}$ $=4k_BT\times BW$, where k_B is the Boltzmann constant, and T is the temperature. This value is to be taken as a lower limit since the photons energy is not sharp but actually distributed (i.e., Poisson).

Non-radiative recombination (NRR) due to the generation of defects and Auger recombination effects may decrease the internal quantum efficiency and influence the final photocurrent output. The current density considering non-radiative recombination effects is evaluated by [5]

$$J_{nr} = qh(A_{nr}n_c + C_n n_c^3) = qh\left(\frac{v_s S_a}{V_a} \cdot n_c + C_n n_c^3\right) \quad (27)$$

where C_n is the Auger recombination coefficient, n_c is the generated carrier density. The generated injected primary photocurrent, I_{ph}, is defined as $I_{ph} = \eta q \cdot P_{opt}/(h_o \nu)$, P_{opt} is the optical power of the injected light, η is the fraction of photons creating electron-hole pairs (i.e., external quantum efficiency), h_o is the Planck's constant, and ν is the light frequency. When considering the non-radiative recombination effect, the realistic photocurrent, ΔI, is

$$\Delta I = (I_{ph} - I_{nr}) \cdot \frac{\tau_n}{\tau_t}, \quad (28)$$

where I_{nr} is the generated current due to the NRR effects, and $I_{nr} = J_{nr} \cdot wh$. τ_n is the electron-hole recombination time, which can be estimated by [5]

$$\tau_n = \frac{\Delta n_c}{g_{ph}}, \quad (29)$$

If a constant light intensity is assumed, where Δn_c is the excess carrier concentration, and g_{ph} is the net optical generation rate, which is related to the photon flux per unit volume by $g_{ph} = \eta \frac{P_{opt}/(h_o \nu)}{whl}$. τ_n is thus re-written by,

$$\tau_n = \frac{\Delta n_c \cdot h_o c \cdot whl}{\eta P_{opt} \lambda_R}, \quad (30)$$

τ_t shown in Eqn. 28 is the transit time of electrons in PD device, and $\tau_t = l/(\mu_n E) = l^2/(\mu_n V)$, μ_n is the electron mobility. Note, the hole mobility μ_p is neglected here due to $\mu_n \gg \mu_p$. The corresponding responsivity is evaluated via $(Current)_{phot}/(Power)_{min}$, which is further written by

$$Responsivity = \frac{\Delta I}{(Power)_{min}} = \frac{\Delta I}{P_{opt}} = \frac{q \Delta n_c \cdot wh}{4k_B T \cdot BW}\left(\frac{\mu_n V}{l}\right)\left[1 - \frac{h_o c \cdot wl(v_s \cdot n_c + C_n n_c^3 h)}{4\eta \lambda_R k_B T \cdot BW}\right] \quad (31)$$

This equation denotes that a high responsivity of a photodetector can be realized by an improved quality material such as withstanding high electric field (i.e., V/l), high mobility, and low surface recombination velocity (main text, Fig. 3).

2.6 *Photodetector Response Speed*

There are several factors that influence the response time of a photodiode and its output circuitry, for instance, transit and diffusion time of photocarriers inside and outside the depletion region, and the detector RC time constant. Here we determine a Sinusoidal steady-state response to represent the response speed [5]. If the optical input power is modulated by a sinusoidal signal, such as

$$P(t)=P_{opt}[1+m\cdot\cos(\omega t)], \tag{32}$$

where m is the modulation index, and m=1 in this work. We achieve the photocurrent response by

$$I\,(t)=I_p\left[1+\frac{m}{\sqrt{1+\omega^2\tau_n^2}}\cos(\omega t-\phi)\right], \tag{33}$$

where $I_p=q\eta\frac{P_{opt}}{h_o v}\frac{\tau_n}{\tau_t}$. Here we use a root-mean-square (rms) optical power, which is $P_{rms}=mP_{opt}/\sqrt{2}$. The *rms* photocurrent signal can be further expressed by [5]

$$i_p=\eta q\frac{P_{rms}}{h_o v}\cdot(\frac{\tau_n}{\tau_t})\frac{1}{\sqrt{1+\omega^2\tau_n^2}} \tag{34}$$

Similar to the case of modulation speed for a laser device, i.e., $H(\omega)/H(0)=1/2$, the 3 dB role-off response can be written by $\frac{i_p(\omega)}{i_p(0)}=1/2$. Using Eqn. 30, the photodetector response f_{3dB} is further given by

$$f_{3dB}=\frac{\sqrt{3}}{2\pi\cdot\tau_n}=\frac{\sqrt{3}}{2\pi}\cdot\frac{\eta\lambda_R P_{opt}}{\Delta n_c h_o c\cdot whl}. \tag{35}$$

This denotes that the response speed is inversely proportional to the device volume.

Toward scaling the responsivity and response speed for PD device, the quantum efficiency, η, may depend on a scaling parameter, which is considered as the intrinsic quantum efficiency, η_i, multiplied the coupling efficiency, $\gamma_{coupling}$, and the absorbance. Here we will introduce the corresponding formulas for the three cavities. For the RR based PD, we take η as a function of ring radius,

$$\eta(r)=\eta_i\cdot\gamma_{coupling\_ring}[1-e^{-\frac{\alpha(r)\Gamma Q(r)\lambda_R}{2\cdot n_g}}], \tag{36}$$

where $\gamma_{coupling\_ring}$ is the coupling efficiency of a ring based PD, n_g is the group index. For the FP cavity based PD, we have η as a function of cavity length,

$$\eta(l)=\eta_i(1-R)(1-e^{-T_{loss}(l)\Gamma(l)}), \tag{37}$$

where R is the mirror reflectivity, and $R=\left|(\frac{\tilde{n}_M-\tilde{n}_D}{\tilde{n}_M+\tilde{n}_D})^2\right|$, $\tilde{n}_M$ and $\tilde{n}_D$ are the complex indices of metal mirror and dielectric material in the cavity, respectively. For the metal nanoparticles enhanced PD, we use

$$\eta(a)=\eta_i\cdot\gamma_{coupling\_MNP}\left[1-e^{-(\eta_{abs}+\eta_{sca})}\right], \tag{38}$$

where $\gamma_{coupling\_MNP}$ is the coupling efficiency of a metal nanoparticle plasmon PD, η_{abs} and η_{sca} are the absorption and scattering efficiencies by retaining only the dipolar modes, respectively, and $\eta_{abs}=\frac{8\pi a}{\lambda}Im(\frac{\varepsilon-1}{\varepsilon+2})$, $\eta_{sca}=\frac{128\pi^4 a^4}{3\lambda^4}\left|\frac{\varepsilon-1}{\varepsilon+2}\right|^2$ [17]. The

corresponding cavity enhanced PD's responsivity and response speed can utilize each cavity's η formula for the device scaling.

3. Link Analysis

In this link analysis section, we benchmarked four different kinds of link options and compared them with conventional electrical link under 22 nm technology node. The first three links are based on each cavity that we analyzed respectively. However, the fourth one is a hybridization that combines metal nanoparticle active devices with passive photonic SOI waveguide, which shows a new trend of recent research in the optical communication field [18]. In this hybrid link, couplers are also need to be considered for plasmonic-photonic conversion. However, even with additional coupling loses, the overall performance should still be better than other link options under certain operating conditions, since it has a fast device speed with low transmission loss (main text, Fig. 5).

$$FOM_{Link} = \frac{Speed}{Energy\ efficiency \times Footprint} = \frac{Capacity}{Energy/bit \times (Scaling \times Distance)} \quad (39)$$

3.1 Link Operating Speed

As we analyzed in the previous section, each cavity has a different speed range as a function of scaling length for lasers, EOMs and photodetectors. However, we assume the entire link operating speed is limited by the slowest device on the link; the rationale is that if this speed is set to be higher than any one of the devices, a buffer has to be set in front of this device, thus introducing higher complexity to the link model. The capacity (i.e., 'speed') for each link option can be calculated based on the Shannon theory [19]

$$'Speed' = Capacity = B \times log_2(1 + \frac{S}{N}) \quad (40)$$

where C represents the link bit rate, whereas the bandwidth B is determined by the slowest device as we explained before. S/N is the signal-to-noise ratio (SNR, in linear scale) and affected by the noise level of the channel. With an appropriate SNR, the bit rate of a single link can be higher than the link operating speed. As a baseline, the link speed SNR equals to unity, when the signal power equals to the noise power, which can be considered a worst case.

By assuming a certain demanded bit error rate (BER), which represents the probability of a bit that received incorrectly, the SNR of the channel can be calculated based on the noise model and coding strategy. Here, we assume on-off-keying (OOK) with "light on" for "1" and "light off" for "0" and the BER to be 10^{-12}, which was recently reported to be an adequate value for on-chip communication [20]. Using a Gaussian noise model, μ represents the mean value of the model which is also the symmetry axis and the σ describes how concentrated these distributions are. With $\mu = 0$ and $\mu = \frac{I_{min}}{Responsivity}$ for transmitted "0" and "1", the goal is to find the appropriate σ to match the chosen BER. Based on the theorem on total probability, the bit error rate should equal to the

probability that receiving a "1" when sending no light and a "0" when sending light (Eqn. 41). Therefore, the variance σ can be derived from the error function expression of the cumulative distribution function (CDF) of a normal distribution for the various photodetector responsivities (Eqn. 42). Next, since the square of the Gaussian distribution variance is just the power of that noise signal, the SNR could be calculated based on Equation 43 with the minimum current requirement of the next stage that this link is connecting and the responsivity of the photodetector. In this way, the SNR for each link as a function of scaling (i.e., responsivity) can be also calculated, which helps increasing the link operating speed. Moreover, the noise model considered here can be regarded as the ideal noise without any thermal noises, shot noises, waveguide crosstalk or other noises such as from fabrications caused in a real application. Thus, the SNR shown here can be regarded as the upper limit.

$$BER = P(0)\times P(1|light\ off) + P(1)\times P(0|light\ on) = 1 - \Phi(0.5\mu/\sigma) = 10^{-12} \quad (41)$$

$$CDF = \Phi\left(\frac{x-\mu}{\sigma}\right) = \frac{1}{2}[1 + erf\ (\frac{x-\mu}{\sigma\sqrt{2}})] \quad (42)$$

$$SNR = \frac{P_{signal}}{P_{noise}} = \frac{I_{min}}{Responsivity\times\sigma^2} \quad (43)$$

3.2 Link Energy Consumption

The energy consumption is another critical factor that will affect the overall performance of each link option. It includes two parts, the laser energy and the modulator energy. The EOM energy consumption has already been calculated in Figure 3c of the main text. However, the energy for the laser is different from the power we calculated in Figure 3a since itis just the threshold power to pump the laser device. In order to obtain a more accurate result, we calculate the laser power based on the responsivity of the photodetectors and the entire link loss, which includes the waveguide loss, impedance mismatching and the insertion loss of the EOMs:

$$'Energy' = Energy\ Efficiency_{laser} = \frac{I_{min}}{WPE\times C\times Responsivity}\times 10^{\frac{|loss|}{10}} \quad (44)$$

where I_{min} represents the minimum current requirement, WPE is the wall-plug efficiency of the laser and *C* is still the link bit rate that derived from Equation 39. As part of the link *loss*, the loss for SOI and metal embedded dielectric waveguides are considered as 1 dB/cm and 0.044 dB/μm, respectively [21, 22]. The photonic-plasmonic coupling loss (mismatch) is regarded as 1 dB based on Ye, C., et al. [23-28], but could be further improved in general.

3.3 Link Scaling and Footprint

We have two parameters for the link area consideration, scaling and footprint. The scaling denotes the 1-dimensional scale of the devices used in the link (main text, Fig. 1); while the footprint has the link propagation distance as the second dimension. Though

footprint is usually the most common metric to judge the on-chip area efficiency, whereas scaling is the other one that directly is related to the device size, thus cannot be ignored. Moreover, the footprint itself is not able to represent the actual device dimensions. For instance, a link with larger scaling devices but short propagation distance may have the same area as another link with tiny scaling devices but much longer distance. Therefore, we consider the Speed/(Energy·Footprint)(main text, Fig. 5), Also, the bit flow density (BFD), which is defined as the total number of bits that can be transferred through given cross-sectional area, could also be considered for these technology options at network level. Within a given chip area, a network that consists of multiple channels, the area model should also consider the actual device area and arrangement, contact pads size, waveguide width with crosstalk pitch for more accurate results.

References

[1] Yariv, A. Critical coupling and its control in optical waveguide-ring resonator systems, *IEEE Photon. Tech., Lett.* **14**, 483-485(2002).

[2] Kiyat, I. and Aydinli, A. High-Q silicon-on-insulator optical rib waveguide racetrack resonators. *Opt. Express*, **13**, 1900-1905 (2005).

[3] Neelakanta, P.S. *Handbook of Electromagnetic Materials: Monolithic and Composite Versions and Their Applications* (CRC Press, Boca Raton, Florida, USA, 1995).

[4] https://courses.cit.cornell.edu/ece533/Lectures/handout11.pdf.

[5] Chuang, S.L. *Physics of Optoelectronic Devices* (Wiley, New York, USA, 1995).

[6] Sun, G., Khurgin, J.B. & Soref, R.A. Plasmonic light-emission enhancement with isolated metal nanoparticles and their coupled arrays. *J. Opt. Soc. Am. B* **25**, 1748-1755 (2008).

[7] Maier, S. Plasmonic field enhancement and SERS in the effective mode volume picture. *Opt. Express*. **14**, 1957-1964 (2006).

[8] Ma, R.M., Oulton, R.F., Sorger, V.J. & Zhang X. Plasmon lasers: coherent light source at molecular scales. *Laser Photon. Rev*. **7**, 1-21 (2013).

[9] Scott, D.B., Chapman, R.A., Wei, C.C, Mahant-Shetti, S.S., Haken, R.A. & Holloway, T.C. Titanium disilicide contact resistivity and its impact on 1-μm CMOS circuit performance, *IEEE Trans. Electron Devices* **34**, 562-574 (1987).

[10] Baraskara, A.K., *et al.* Ultralow resistance, nonalloyed Ohmic contacts to n-InGaAs. *J. Vac. Sci. Technol. B* 27, 2036-2039 (2009).

[11]Egglestona, M.S., Messera, K., Zhang, L., Yablonovitch, E. & Wu, M.C. Optical antenna enhanced spontaneous emission. *Proc. Natl. Acad. Sci*. USA **112**, 1704-1709 (2015).

[12]Fekecs, A., et al. Fabrication of high resistivity cold-implanted InGaAsP photoconductors for efficient pulsed terahertz devices. *Opt. Mater. Express* **1**, 1165-1177 (2011)

[13] Genov, D.A., Oulton, R.F., Bartal, G. & Zhang X. Anomalous spectral scaling of light emission rates in low-dimensional metallic nanostructures. *Phy. Rev. B* **83**, 245312 (2011).

[14] Miller, D.A.B. Energy consumption in optical modulators for interconnects. *Opt. Express* **20**, A293-A308 (2012).

[15] Lin, H.T., Ogbuu, O., Liu, J., Zhang, L., Michel, J. & Hu, J.J. Breaking the energy-bandwidth limit of electrooptic modulators: theory and a device proposal. *J. Lightwave Technol.* **31**, 4029-4036 (2013).

[16] Li, G., *et al*. Ring resonator modulators in Silicon for interchip photonic links. *IEEE J. Sel. Top. Quantum Electro*. **19**, 3401819 (2013).

[17] Akimov, Yu. A., Ostrikov, K., & Li, E.P. Surface plasmon enhancement of optical absorption in thin-film Silicon solar cells. *Plasmonics* **4**,107-113 (2009).

[18] Sun, S., Badawy, A.H.A., Narayana, V., El-Ghazawi, T. & Sorger, V.J. The case for hybrid photonic plasmonic interconnects (HyPPIs): Low-latency energy-and-area-efficient on-chip interconnects. *IEEE Photonics J.* **7**, 1-14 (2015).

[19] Shannon, C.E. A mathematical theory of communication. *ACM SIGMOBILE Mobile Computing and Communications Review*, **5**, 3-55 (2001).

[20] Majumdar, A., Cunningham, J.E. & Krishnamoorthy, A.V. Alignment and performance considerations for capacitive, inductive, and optical proximity communication. *IEEE Trans. Adv. Packag*. **33**, 690-701 (2010).

[21] Li, G.L. *et al.* Ultralow-loss, high-density SOI optical waveguide routing for macrochip interconnects. *Opt. Express*. **20**, 12035-12039 (2012).

[22] Berini, P. & De Leon, I. Surface plasmon-polariton amplifiers and lasers. *Nature Photon*. **6**, 16-24 (2012).

[23] Ye, C., Khan, S., Li, Z.R., Simsek, E. & Sorger, V.J. λ-size ITO and graphene-based electro-optic modulators on SOI. *IEEE J. Sel. Top. Quantum Electron.* **20**, 40-49 (2014).

[24] Ye, C., Liu, K., Soref, R.A. & Sorger, V.J. A compact plasmonic MOS-based 2×2 electro-optic switch. *Nanophotonics* **4**, 261-268 (2015).

[25] Huang, C., Lamond, R.J., Pickus, S.K., Li, Z.R. & Sorger, V.J. A Sub-size modulator beyond the efficiency-loss limit. *IEEE Photonics J.* **5**, 2202411 (2013).

[26] Ma, Z.Z., Li, Z.R., Liu, K., Ye, C.R. & Sorger, V.J. Indium tin oxide for high performance electro-optic modulation. *Nanophotonics* **4**, 198-213 (2015).

[27] Sorger, V. J. Nano-optics gets practical plasmon modulators. *Nat. Nanotechnol*. **10**, 11-15 (2015).

[28] Liu, K., Ye, C.R., Khan, S. & Sorger, V.J. Review and perspective on ultrafast wavelength size electro-optic modulators. *Laser Photon. Rev*. **9**, 172-194 (2015).
